# Impaired Nuclear Export of Polyglutamine-Expanded Androgen Receptor in Spinal and Bulbar Muscular Atrophy

**DOI:** 10.1038/s41598-018-36784-4

**Published:** 2019-01-15

**Authors:** Frederick J. Arnold, Anna Pluciennik, Diane E. Merry

**Affiliations:** Department of Biochemistry and Molecular Biology, Thomas Jefferson University, Philadelphia, PA 19107 USA

## Abstract

Spinal and bulbar muscular atrophy (SBMA) is a neuromuscular disease caused by polyglutamine (polyQ) expansion in the androgen receptor (AR). Prior studies have highlighted the importance of AR nuclear localization in SBMA pathogenesis; therefore, in this study, we sought to determine the role of AR nuclear export in the pathological manifestations of SBMA. We demonstrate here that the nuclear export of polyQ-expanded AR is impaired, even prior to the formation of intranuclear inclusions of aggregated AR. Additionally, we find that promoting AR export with an exogenous nuclear export signal substantially reduces its aggregation and blocks hormone-induced toxicity. Moreover, we show that these protective effects are conferred by destabilization of the mutant protein due to an increase in proteasomal degradation of the cytoplasmic AR. Despite a growing body of evidence that global disruption of nucleo/cytoplasmic transport occurs in ALS and HD, our data suggest that no such global disruption occurs in models of SBMA; rather, AR-specific mechanisms, including reduced phosphorylation at Serine 650, are likely responsible for the impaired nuclear export of polyQ-expanded AR.

## Introduction

Spinal and bulbar muscular atrophy (SBMA) is an X-linked neuromuscular disease caused by a polyglutamine-encoding CAG trinucleotide repeat expansion in the first exon of the androgen receptor gene^1^. SBMA affects approximately 1 in 40,000 males^2^, with onset of neurologic symptoms typically occurring between 30 and 50 years of age^3,4^. As in other CAG trinucleotide repeat disorders, such as Huntington’s Disease (HD), longer CAG repeat tracts are associated with earlier disease onset^3,5,6^.

SBMA is characterized by progressive degeneration of lower motor neurons in the brain stem and spinal cord, which, along with a primary myopathy^7^, causes weakness and cramping of the muscles, dysphagia, dysarthria, and immobility^2,3,8–11^. Additionally, progressive loss of the bulbar musculature predisposes patients to potentially fatal aspiration-induced pneumonia. While SBMA patients show signs of androgen insensitivity, including gynecomastia and reduced fertility, the neurologic symptoms of SBMA are not caused by a loss of AR function, as neuromuscular symptoms are not observed in patients with complete androgen insensitivity syndrome^12^. Rather, SBMA is caused by a toxic property of polyQ-expanded AR, as further evidenced by the fact that expression of polyQ-expanded AR in the presence of ligand is sufficient to cause neuromuscular symptoms in mouse models^13–16^ and toxicity in cultured cells^17^. Moreover, while evidence from a Drosophila model of SBMA suggests that SBMA arises from a gain of native transcriptional activities^18^, findings from a knock-in mouse model of SBMA suggest that enhancing AR transcriptional activity can be protective^19^.

The AR is a steroid hormone receptor that resides in the cytoplasm, in the absence of ligand, in an inactive aporeceptor complex that contains chaperones (Hsc70, Hsp40, Hsp90, HIP, HOP), p23, and immunophilins (Cyp40, FKBP51, FKBP52)^20,21^. Upon binding of testosterone or 5α-dihydrotestosterone (DHT), the AR undergoes a conformational change, inducing its nuclear localization and transcriptional regulation of target genes. There is evidence that the AR is exported from the nucleus and degraded in the cytoplasm by the ubiquitin-proteasome system^22–26^.

In contrast with other aspects of AR metabolism, little is known about the mechanism of AR nuclear export. Mutagenesis experiments identified a 75-amino acid region (residues 743–817) in the AR ligand binding domain (LBD) that is both necessary and sufficient for AR nuclear export in a PC3 prostate cancer cell model^27^. The putative nuclear export signal (NES) contained within this region is Leptomycin B-insensitive, suggesting that this regulatory motif is not a candidate for chromosomal maintenance 1 (CRM1) – mediated nuclear export upon hormone withdrawal^23,27,28^. However, a putative CRM1 binding site was identified near the C-terminus of the AR, and the rapid nuclear export of AR that occurs upon inhibition of GSK-3β in prostate cancer cells is blocked by Leptomycin B^29,30^. Thus, while AR undergoes slow, Leptomycin B-insensitive export upon hormone withdrawal, it may undergo rapid, CRM1-mediated nuclear export depending on the physiological conditions of the cell^29^. Interestingly, a similar phenomenon has been observed in the nuclear export of the glucocorticoid receptor (GR), a member of the nuclear receptor superfamily with a high degree of homology to the AR. While GR export induced by ligand withdrawal is slow and Leptomycin B-insensitive, GR undergoes rapid, Leptomycin-B sensitive nuclear export regulated by c-JUN N-terminal kinase (JNK) upon UV exposure^31^.

In addition to the NES identified in the AR LBD, residues in both the DNA-binding domain (DBD) and the hinge region also appear to regulate AR nuclear export. A double mutation in the DBD (F582, 583A) is sufficient to block nuclear export; however, DNA binding itself is not required for AR nuclear export, as the DNA-binding mutant V581F does not affect export^32,33^. Additionally, phosphorylation of the AR at serine 650 has been shown to regulate nuclear export of the AR^34,35^. In COS-7 and LNCaP cells, phosphorylation of S650 is mediated by the MAPK kinase 4/JNK and MAPK kinase 6/p38 stress kinase signaling pathways. Inhibition of JNK and p38 reduced the nuclear export of wildtype AR in COS-7 cells to an equivalent extent as a phospho-null (S650A) mutation but had no effect on AR with a phospho-mimic (S650D) mutation^34^. Consistent with these findings, knockdown of MAP3k11, an upstream regulator of JNK activity, decreased S650 phosphorylation in LNCaP and C2-4B cells^36^. Additionally, inhibition of protein phosphatase 1 alpha (PP1α) by tautomycin led to an increase in S650 phosphorylation and a decrease in AR nuclear accumulation in LNCaP cells^35^. Notably, increasing AR nuclear export was associated with a decrease in its transcriptional activity, while blocking export increased AR transcriptional activity^25,32,34,35^.

Nuclear accumulation and aggregation of polyQ disease proteins is a common pathological feature of all nine CAG trinucleotide repeat disorders, highlighting its importance for further study^37^. While the extent to which toxicity in each of these diseases is mediated by nuclear versus cytoplasmic aggregation is not fully understood, targeting polyQ proteins to the nucleus or cytoplasm can modulate aggregation and/or toxicity in several cell and animal models of polyQ disease^17,18,38–42^. In some polyQ diseases, there is already evidence for defective nuclear export of the polyQ disease protein. For example, polyQ-expanded N-terminal huntingtin fragments exhibited a reduced interaction with the nuclear pore protein translocated promoter region (Tpr), increasing their nuclear accumulation^43^. Additionally, nuclear export of both mutant ataxin-3 and mutant ataxin-7 was reduced compared with the respective wildtype proteins^39,44^, while nuclear export of mutant ataxin-1 was completely blocked^45^. Thus, nuclear localization appears to play a key role in the pathogenesis of polyQ disease, with evidence for the therapeutic benefit of decreasing nuclear accumulation of some polyQ disease proteins.

In cell and animal models of SBMA, both the presence of hormone^13,14,16,46^ and the nuclear localization^17,18,46^ of mutant AR are necessary for toxicity and neuromuscular pathology, with the formation of intranuclear inclusions of aggregated AR in neuronal and non-neuronal tissues a hallmark of the disease state^47,48^. Given the requirement for nuclear localization in disease-mediated toxicity, we sought to determine if the nuclear export of polyQ-expanded AR is disrupted and whether enhancing the nuclear export of polyQ-expanded AR would be protective in cell models of SBMA. The experiments presented here represent the first investigation of the role of AR nuclear export in SBMA pathogenesis and offer new insights into the effect of a polyQ-expansion on this important process.

## Results

### PolyQ-expanded AR exhibits reduced nuclear export

**Figure 1:**
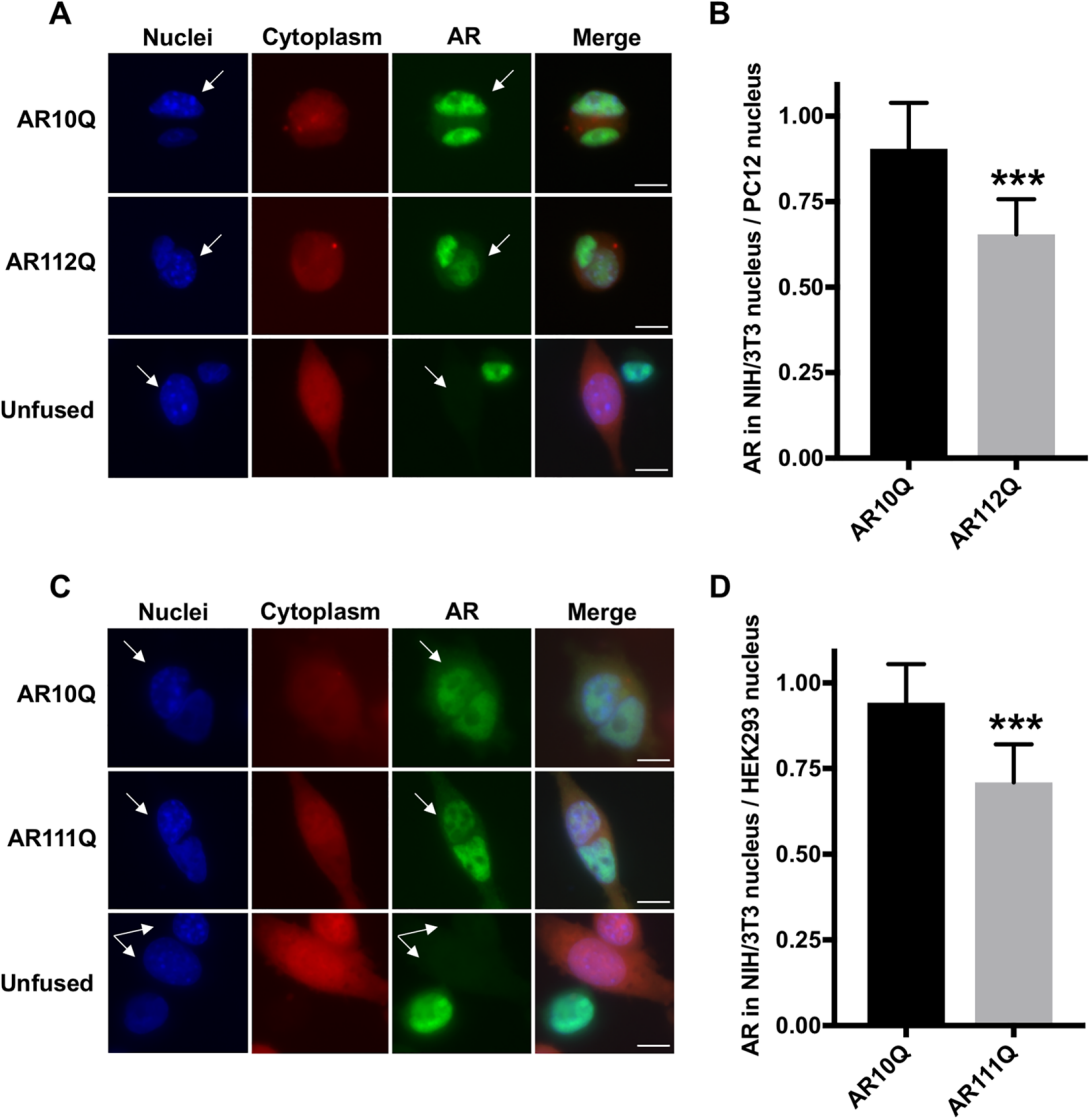
PolyQ-expanded AR exhibits reduced nuclear export. (**A**,**C**) Immunofluorescence images of heterokaryons formed by fusing PC12 (**A**) or HEK293 (**C**) cells, inducibly expressing wildtype or polyQ-expanded AR, with AR-negative NIH/3T3 cells and treated with 10 nM DHT for 4 hrs post-fusion. NIH/3T3 nuclei were identified by the presence of bright, condensed chromatin upon Hoechst staining (indicated by white arrows). Heterokaryons were verified as two nuclei surrounded by CellTracker^TM^ Orange CMTMR dye with AR in at least one nucleus. (**B**,**D**) Quantification of images from (**A**,**C**) was performed by measuring the fluorescence intensity of AR in the acceptor (NIH/3T3) nucleus relative to the donor (PC12/HEK293) nucleus in each heterokaryon. 20–30 heterokaryons were analyzed per condition. Statistical significance was determined by Student’s t-test. ***p < 0.001. Experiment (**A**,**B**) was repeated five times. Experiment (**C**,**D**) was repeated three times. Error bars = SD. Scale bars represent 10 μm.

To compare the nuclear export of polyQ-expanded AR with wildtype AR, we utilized a well-characterized PC12 cell model of SBMA in which rat pheochromocytoma-derived PC12 cells express full-length human AR under the control of a tetracycline-inducible promoter^17,19,49–55^. Using PC12 cells expressing AR10Q (wildtype) or AR112Q (polyQ-expanded), we performed a heterokaryon shuttling assay, a technique previously used to measure the nuclear export of wildtype AR^32–34^. Briefly, AR expression was induced in PC12 cells for 24 hrs with doxycycline (DOX). Following DOX washout, the cells were treated for 2 hrs with DHT to induce AR nuclear translocation and with cycloheximide to block protein synthesis from any remaining AR mRNA. Separately, NIH/3T3 mouse fibroblast cells, which lack AR expression, were marked by staining with CellTracker^TM^ Orange CMTMR Dye, a diffuse cytoplasmic marker that converts to a membrane-impermeable form after cell uptake. These two cell populations were then fused, generating heterokaryons, i.e., cells containing both PC12 and NIH/3T3 nuclei within a shared cytoplasm. Heterokaryons were then treated with DHT and cycloheximide for an additional 4 hrs to allow nucleocytoplasmic transport to occur between the PC12 (donor) and NIH/3T3 (acceptor) nuclei. While some soluble AR oligomeric species were detected by SDS-agarose gel electrophoresis (SDS-AGE) within this timeframe (Fig. S1), intranuclear inclusions of AR112Q were not observed. Moreover, the nuclear import of AR10Q and AR112Q was previously shown to occur with comparable kinetics^19^. Following immunohistochemistry, we analyzed heterokaryons by quantifying the amount of AR that translocated to the NIH/3T3 nucleus and the amount of AR that remained in the PC12 nucleus. This analysis revealed that, while AR10Q was evenly distributed between the PC12 and NIH/3T3 nuclei, AR112Q was retained to a greater extent within the PC12 nuclei, indicating deficient nuclear export of polyQ-expanded AR (Fig. 1A,B).

To further validate the finding that reduced nuclear export of polyQ-expanded AR occurred prior to intranuclear inclusion formation, we assessed AR nuclear export in a second cell model of SBMA. In isogenic, Flp-In HEK293 cells inducibly expressing full-length human AR10Q or AR111Q under the control of a tetracycline-inducible promoter, the heterokaryon shuttling assay revealed that AR111Q was retained in the HEK293 nucleus of analyzed heterokaryons, while AR10Q was evenly distributed between the HEK293 and NIH/3T3 nuclei (Fig. 1C,D). As in PC12 cells, soluble AR aggregation species were detected in HEK293 cells expressing polyQ-expanded AR (Fig. S1); however, intranuclear inclusions of AR111Q were not observed until two weeks of treatment with DHT (data not shown). These results demonstrate that the nuclear export of polyQ-expanded AR is impaired and that this impairment is not caused by the accumulation of mutant AR into insoluble intranuclear inclusions.

### The addition of an exogenous NES reduces the aggregation and hormone-induced toxicity of polyQ-expanded AR

**Figure 2:**
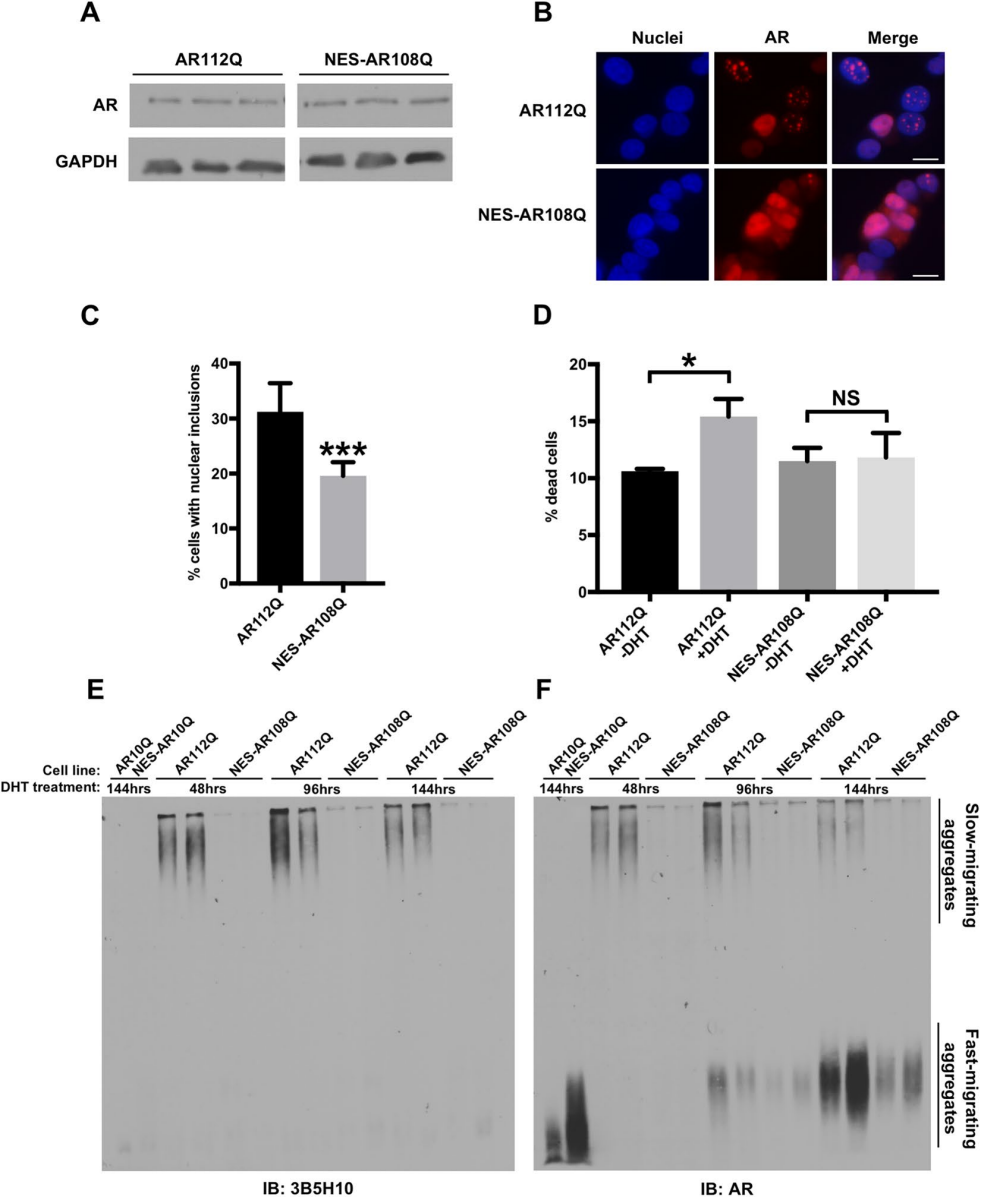
The addition of an exogenous NES reduces the aggregation and hormone-induced toxicity of polyQ-expanded AR. (**A**) Western analysis of PC12 cells, expressing AR112Q or NES-AR108Q, treated with DOX for 24 hrs, confirmed equivalent expression AR112Q and NES-AR108Q. (**B**) Immunofluorescence images of PC12 cells expressing AR112Q or NES-AR108Q following 72 hrs treatment with 10 nM DHT. Scale bars represent 10 μm. (**C**) Percentage of PC12 cells expressing AR112Q or NES-AR108Q with nuclear inclusions following 72 hrs treatment with 10 nM DHT. 300–350 cells were counted in triplicate per condition. Statistical significance was determined by Student’s t-test. ***p < 0.001. (**D**) PC12 cells expressing AR112Q or NES-AR108Q were treated with 10 nM DHT or ethanol (vehicle) for 12 days. Dead cells were identified via trypan blue staining. 200–250 cells were counted in triplicate per condition. Statistical significance was determined by one-way ANOVA with *post hoc* Tukey test. *p < 0.05. (**E**,**F**) Western analysis of PC12 cell lysates resolved by SDS-agarose gel electrophoresis following treatment with 10 nM DHT for the indicated times. At each time point, NES-AR108Q formed fewer aggregation species than AR112Q as detected by 3B5H10 (**E**) and AR (**F**) antibodies. Note that the signal in the AR10Q and NES-AR10Q lanes represents a monomeric form of AR10Q. Experiments were repeated three times. Error bars = SD.

Having established that polyQ-expanded AR exhibits impaired nuclear export, we next wanted to determine if this aspect of AR metabolism plays a role in the aggregation and toxicity of mutant AR. Previous studies in several cell and animal models of SBMA have highlighted the requirement of AR nuclear localization for disease^17,18,46^. Thus, we hypothesized that increasing the nuclear export of polyQ-expanded AR would be protective in SBMA models. To test this hypothesis, we cloned the previously described NES of cAMP-dependent protein kinase inhibitor alpha (MLALKLAGLDI)^56^ to the N-terminus of the AR and generated a new, stably transfected clonal PC12 cell line expressing NES-AR108Q. As shown in Fig. 2, NES-AR108Q displayed increased distribution to the cytoplasm of PC12 cells in the presence of hormone, compared with equivalently expressed AR112Q (Figs 2A,B, S2A, quantified in Fig. S2B). In the absence of hormone, the localization of mutant AR was unaffected by the exogenous NES (Fig. S2A).

Upon treatment with hormone, PC12 cells expressing polyQ-expanded AR form intranuclear inclusions that resemble the AR inclusion bodies observed in SBMA patient tissues, in that they consist of proteolyzed N-terminal fragments of AR^47–49,55^. The addition of the exogenous NES reduced the frequency of cells containing intranuclear inclusions (Fig. 2B,C). AR aggregation species can be further evaluated by SDS-AGE, an established method for resolving large aggregates of polyQ proteins by agarose gel electrophoresis^57–59^. We have previously identified two distinct populations of AR112Q aggregation species by SDS-AGE, slow-migrating aggregates that contain full-length AR and are SDS-soluble, and fast-migrating aggregates that contain both full-length AR and N-terminal AR fragments, are largely SDS-insoluble, and temporally correlate with intranuclear inclusions^54,55^. Additionally, slow-migrating aggregation species are detected by the 3B5H10 monoclonal antibody, which recognizes expanded polyQ tracts in low-molecular weight oligomers, but not in higher molecular weight inclusion bodies^60^. The 3B5H10 antibody also recognizes monomeric mutant AR^60^ and, consistent with this finding, detects both cytoplasmic and nuclear NES-AR108Q (Fig. S2C). Increasing AR nuclear export reduced the formation of both slow- and fast-migrating aggregation species over time (Fig. 2F). Moreover, NES-AR108Q formed fewer 3B5H10-immunoreactive oligomers (Fig. 2E). These data suggest that aberrant nuclear retention of AR112Q contributes to its aggregation in PC12 cells.

AR expression in PC12 cells also causes polyQ length- and hormone-dependent cell death^17^, allowing us to assess the toxicity of NES-AR108Q in this cell model. We observed that enhancing the nuclear export of polyQ-expanded AR eliminated DHT-induced cell death (Fig. 2D). Altogether, these results demonstrate that increasing the nuclear export of polyQ-expanded AR has a protective effect on phenotypes relevant to SBMA pathogenesis, such as AR aggregation and hormone-induced toxicity, and support the hypothesis that deficient nuclear export of polyQ-expanded AR contributes to disease.

### Enhancing AR nuclear export promotes its proteasomal degradation

**Figure 3:**
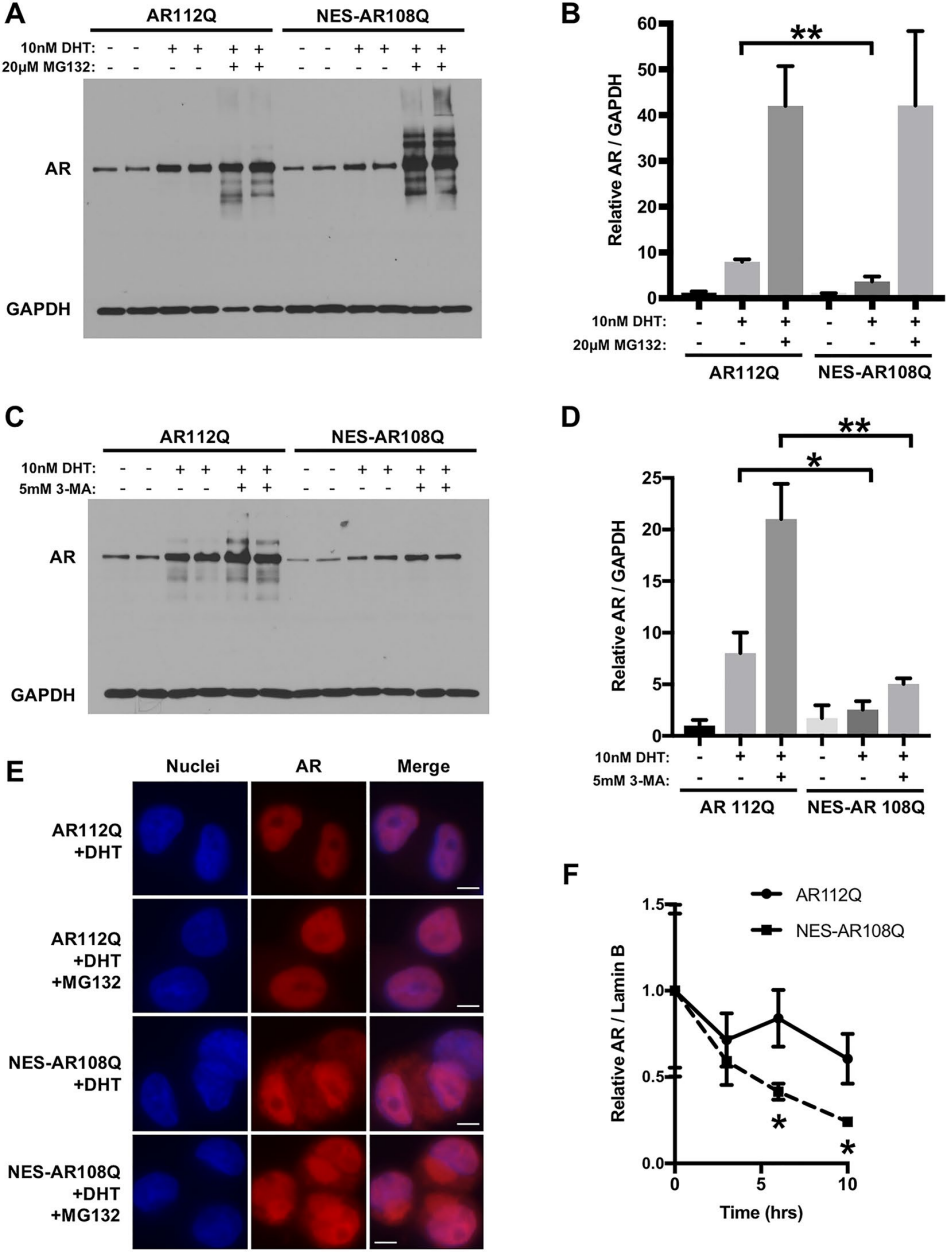
Proteasome-mediated degradation of NES-AR reduces its stabilization by hormone and decreases its half-life. (**A**) PC12 cells expressing AR112Q or NES-AR108Q were treated with DOX for 24 hrs. Following DOX washout, cells were treated for an additional 24 hrs with 10 nM DHT or ethanol (vehicle) and 20 μM MG132 or DMSO (vehicle) and analyzed by Western blot. (**B**) Quantification of (**A**), comparing the protein levels of AR112Q with NES-AR108Q for each condition. Statistical significance for each condition was determined by Student’s t-test. **p < 0.01. (**C**) PC12 cells expressing AR112Q or NES-AR108Q were treated with DOX for 24 hrs. Following DOX washout, cells were treated for an additional 24 hrs with 10 nM DHT or ethanol (vehicle) and 5 mM 3-MA or H_2_O (vehicle) and analyzed by Western blot. (**D**) Quantification of (**C**), comparing the protein levels of AR112Q with NES-AR108Q for each condition. Statistical significance for each condition was determined by Student’s t-test. *p < 0.05, **p < 0.01. (**E**) Immunofluorescence images of PC12 cells expressing AR112Q or NES-AR108Q treated for 24 hrs with 10 nM DHT and 20 μM MG132 or DMSO (vehicle). Scale bars represent 5 μm. (**F**) Determination of AR half-life. PC12 cells expressing AR112Q or NES-AR108Q were treated with DOX for 24 hrs. Following DOX washout, cells were treated with 10 nM DHT for 2 hrs, then with 10 nM DHT and 10 μg/mL cycloheximide for the indicated times. Statistical significance at each time point was determined by Student’s t-test. *p < 0.05. Experiments were repeated three times. Error bars = SD.

In order to understand the mechanism by which increasing the nuclear export of polyQ-expanded AR reduces its aggregation and blocks hormone-induced toxicity, we assessed the stability of NES-AR108Q following DHT treatment. Degradation of wildtype AR by the ubiquitin-proteasome system in the cytoplasm has been widely reported^22–26^, while retaining polyQ-expanded AR in the cytoplasm by mutating its nuclear localization signal (NLS) leads to autophagic AR degradation^17^. Thus, we hypothesized that targeting mutant AR to the cytoplasm with an exogenous NES could increase its degradation by one or both of these pathways. As shown in Fig. 3A–D, the DHT-dependent stabilization of polyQ-expanded AR protein was substantially reduced by addition of the exogenous NES. Furthermore, the half-life of NES-AR108Q was significantly shorter than that of AR112Q, providing additional evidence that enhancing AR nuclear export destabilizes the mutant protein (Figs 3F, S3).

To determine whether the reduction in AR stability was due to increased degradation of NES-AR108Q by the proteasome and/or by autophagy, we assessed the ability of DHT to stabilize NES-AR108Q when each of these pathways was inhibited. Inhibiting the proteasome with MG132 completely restored NES-AR108Q to a level equivalent to AR112Q upon MG132 treatment (Figs 3A,B, S4A), while inhibiting autophagy with 3-methyladenine (3-MA) had little effect on NES-AR 108Q levels (Figs 3C,D, S4B), indicating that the observed decrease in NES-AR108Q stability was a result of its proteasomal degradation. Moreover, in response to proteasomal inhibition, we observed an accumulation of NES-AR108Q in the cytoplasm (Fig. 3E, quantification shown in Fig. S4C). The observed cytoplasmic deposits of NES-AR108Q (and to a lesser extent of AR112Q) colocalized with known markers of aggresomes^61–63^ – aggregates of misfolded proteins that form when the capacity of the proteasome to degrade these proteins is exceeded (such as when the proteasome is inhibited)^64^ (Fig. S5A,B). 3B5H10-immunoreactive species were also found within aggresomes (Fig. S5C), suggesting that the reduced number of NES-AR108Q aggregation species detectable by this antibody on SDS-AGE (Fig. 2E) is the result of enhanced proteasomal degradation of NES-AR108Q in the cytoplasm. Notably, inhibition of autophagy with 3-MA did not lead to the formation of AR-containing aggresomes (Fig. S5D,E). Taken together, these data indicate that targeting polyQ-expanded AR to the cytoplasm reduces its aggregation and ligand-induced toxicity by enhancing its proteasomal degradation.

### Reduced serine 650 phosphorylation contributes to the impaired nuclear export of polyQ-expanded AR

**Figure 4:**
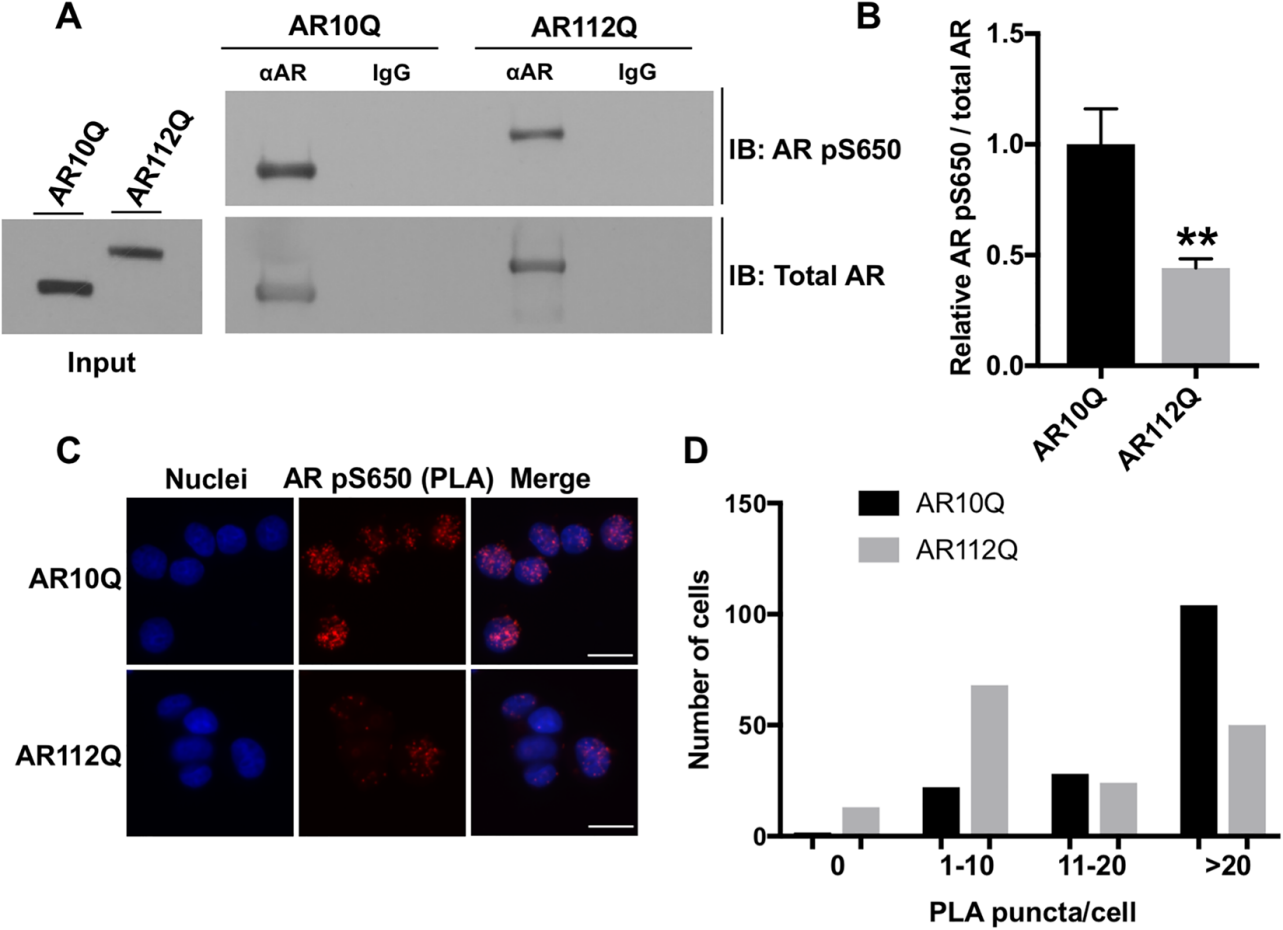
PolyQ-expanded AR exhibits reduced phosphorylation at Serine 650. PC12 cells expressing AR10Q or AR112Q were treated with 10 nM DHT for 24 hrs. (**A**) Immunoprecipitation of total AR followed by immunoblotting with an antibody specific to AR phosphorylated at S650 (pS650) revealed a substantial decrease in polyQ-expanded AR pS650. (**B**) Triplicate samples from (**A**) were quantified by measuring relative levels of AR pS650 versus total AR. Statistical significance was determined by Student’s t test. **p < 0.01. (**C**) Immunofluorescence images of PC12 cells expressing AR10Q or AR112Q following proximity ligation assay using primary antibodies specific for total AR and AR pS650. Scale bars represent 20 μm. (**D**) Quantification of the number of PC12 cells from **C** with 0, 1–10, 11–20, or >20 PLA puncta per cell. 155 cells were counted per condition. ***p < 0.001 as determined by Kolmogorov-Smirnov test. Experiments were repeated three times. Error bars = SD.

The nuclear export of wildtype AR was previously shown to be regulated by phosphorylation of serine 650^34^, a residue within the hinge region of the AR. Having found that polyQ-AR is deficient in its nuclear export by the heterokaryon shuttling assay, we hypothesized that changes in the phosphorylation state of S650 in polyQ-expanded AR could play a role in its impaired nuclear export. Analysis of AR phosphorylation at Serine 650 (pS650) revealed that polyQ-expanded AR exhibits markedly reduced phosphorylation at S650 compared to wildtype AR (Fig. 4A,B). This biochemical analysis was independently confirmed by Proximity Ligation Assay (PLA), a technique that can be used to detect, not only interacting proteins, but protein modifications as well. Using primary antibodies to detect both total AR and AR pS650, the PLA analysis confirmed our biochemical analysis, revealing that polyQ-expanded AR displays reduced phosphorylation at S650 (Fig. 4C,D).

**Figure 5:**
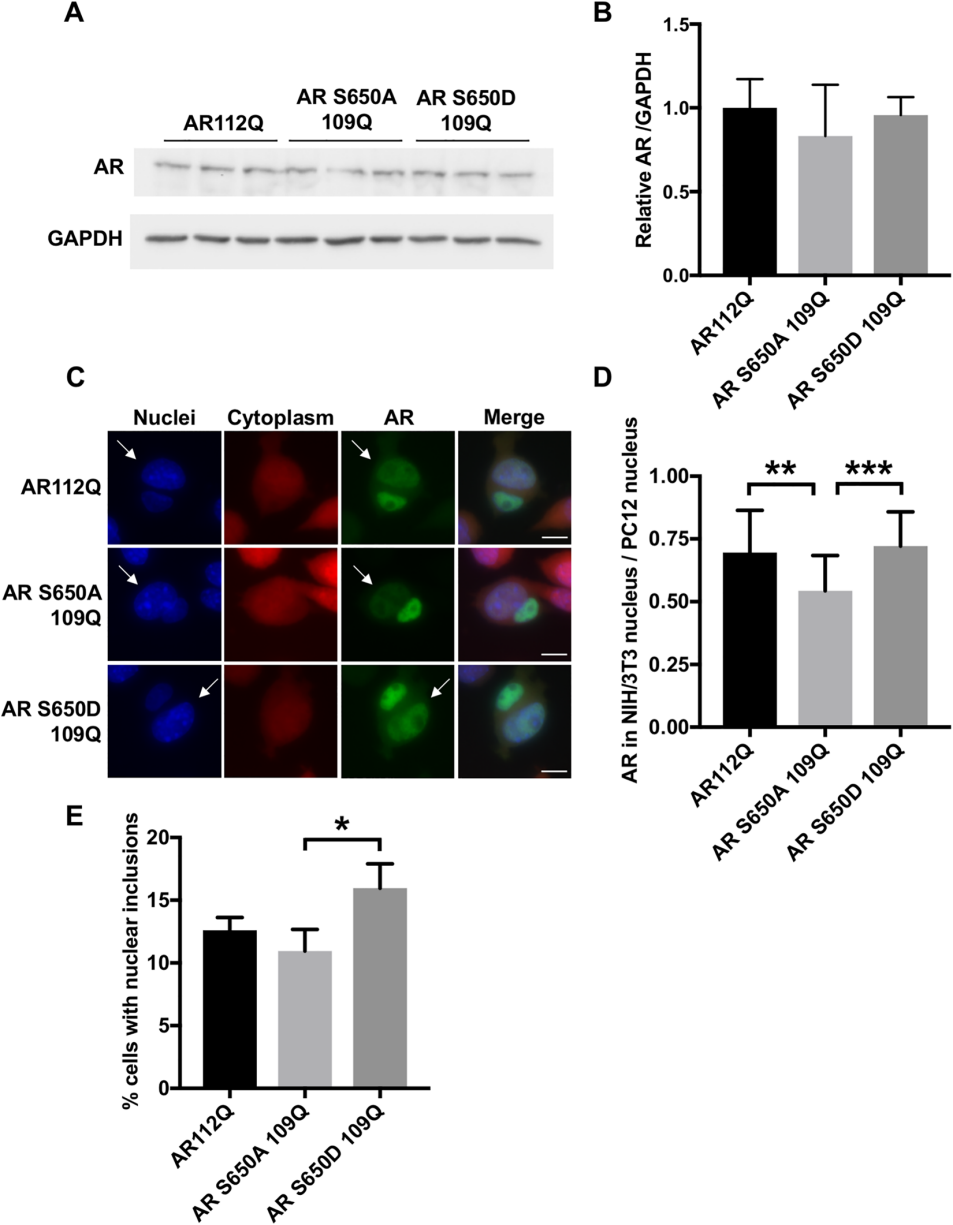
Reduced serine 650 phosphorylation contributes to the impaired nuclear export of polyQ-expanded AR. (**A**,**B**) Western analysis confirmed equivalent expression of AR112Q, AR S650A 109Q, and AR S650D 109Q in clonal PC12 cell lines following 24 hrs of treatment with DOX. (**C**) Heterokaryon analysis revealed that AR S650A 109Q is more highly retained in the donor (PC12) nucleus than the acceptor (NIH/3T3) nucleus (indicated by white arrows). Scale bars represent 10 μm. (**D**) Quantification of the fluorescence intensity of AR in the acceptor nucleus relative to the donor nucleus in each heterokaryon. 20–25 heterokaryons were analyzed per condition. Statistical significance was determined by one-way ANOVA with *post hoc* Tukey test. **p < 0.01, ***p < 0.001. (**E**) Percentage of PC12 cells with nuclear inclusions following 48 hrs treatment with 10 nM DHT. 300–350 cells were counted in triplicate per condition. Statistical significance was determined by one-way ANOVA with *post hoc* Tukey test. *p < 0.05. Experiments were repeated three times. Error bars = SD.

Having established that pS650 is reduced in polyQ-expanded AR, we sought to validate that pS650 regulates nuclear export in PC12 cells, as has been reported in COS-7 cells^34^. New, stably-transfected clonal PC12 cell lines expressing wildtype and polyQ-expanded AR with either phospho-null (S650A) or phospho-mimic (S650D) mutations were established and DOX doses determined to achieve equivalent AR protein levels in the absence of DHT (Fig. 5A). Using the previously described heterokaryon shuttling assay, we assessed the effect of these mutations on AR nuclear export. We observed that mutation of S650 to prevent its phosphorylation (S650A) further impaired the nuclear export of polyQ-expanded AR (Fig. 5C,D), suggesting that reduced pS650 may contribute to its nuclear export deficiency. An effect of S650A on the nuclear export of wildtype AR was not observed (Fig. S6A,B).

To investigate the role of S650 phosphorylation in AR aggregation, we compared the frequency of intranuclear inclusions of AR112Q, AR S650A 109Q, and AR S650D 109Q in PC12 cells. We found that the phospho-null mutation, S650A, did not further increase intranuclear inclusion frequency of polyQ-expanded AR, while the S650D mutation actually increased AR aggregation relative to AR S650A 109Q (Fig. 5E).

Altogether, these experiments demonstrate that S650 modulates two aspects of AR metabolism relevant to SBMA pathogenesis - aggregation and nuclear retention. We found that the phosphorylation of polyQ-expanded AR at S650 was reduced, and that blocking S650 phosphorylation exacerbated the nuclear export deficit of polyQ-expanded AR. Additionally, we found that the S650D mutation increased AR intranuclear inclusions without affecting its nuclear retention. The mechanism by which S650 regulates these processes remains to be explored.

### Inhibition of GSK-3β does not promote AR nuclear export in PC12 or HEK293 cells

**Figure 6:**
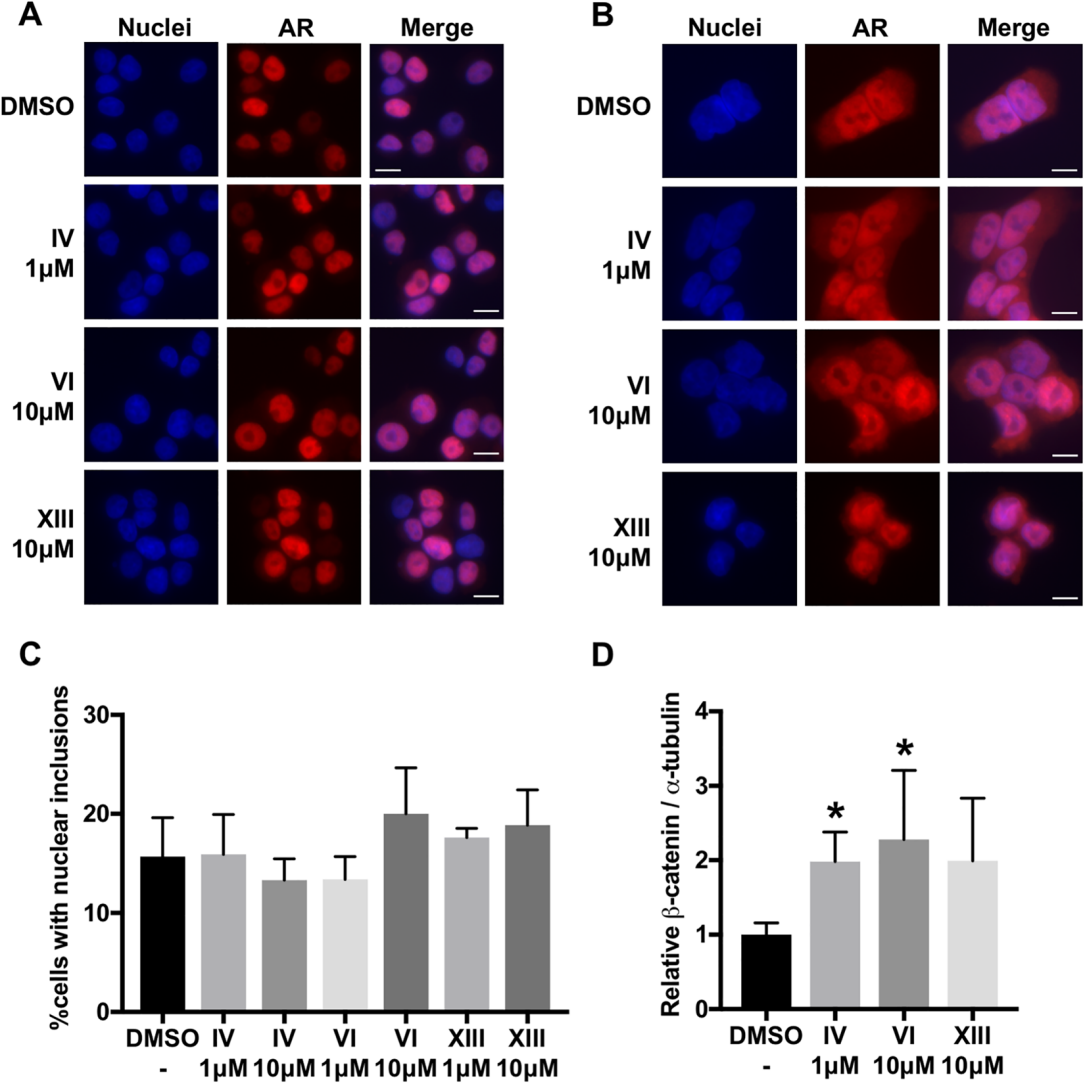
Inhibition of GSK-3β does not promote AR nuclear export in PC12 or HEK293 cells. Immunofluorescence images of PC12 (**A**) and HEK293 (**B**) cells expressing AR10Q treated for 30 min with 10 nM DHT then 4 hrs with 10 nM DHT plus GSK-3 inhibitor IV, GSK-3β inhibitor VI, GSK-3 inhibitor XIII, or DMSO (vehicle) at the indicated doses. Scale bars represent 10 μm. (**C**) Percentage of PC12 cells with nuclear inclusions following 24 hrs treatment with 10 nM DHT then 24 hrs with 10 nM DHT plus GSK-3β inhibitors or DMSO (vehicle) at the indicated doses. 300–350 cells were counted in triplicate per condition. (**D**) Quantitative analysis of GSK-3β inhibitor efficacy on the stabilization of β-catenin, as previously reported^72^. Western analysis of HEK293 cells expressing AR10Q treated with GSK-3 inhibitor IV, GSK-3β inhibitor VI, or DMSO (vehicle) for 48 hrs or with GSK-3 inhibitor XIII for 24 hrs (shortened treatment due to toxicity of the compound). Western blot shown in Fig. S7. Statistical significance was determined by pairwise comparison of each treatment with the control by one-tailed Student’s t test. *p < 0.05. Experiments (**A**), (**B**) and (**C**) were repeated three times. Error bars = SD.

In addition to the slow, CRM1-independent nuclear export of AR observed after hormone withdrawal, it has been proposed that the AR undergoes rapid, CRM1-dependent nuclear export upon inhibition of GSK-3β^29,30,65^. GSK-3β is known to phosphorylate and negatively regulate the transcriptional activity of the AR as well as other steroid hormone receptors, supporting the notion that the effect of GSK-3β inhibition on AR function may be conserved across steroid hormone receptors^29,30,65–71^. Despite evidence that inhibition of GSK-3β causes rapid nuclear export of endogenous and exogenous AR in multiple prostate cancer cell lines, as well as confirmation that the GSK-3β inhibitors used here had the expected effect of stabilizing β-catenin levels^72^ (Fig. 6D), GSK-3β inhibition had no effect on the subcellular localization of wildtype or mutant AR in PC12 or HEK293 cells (Fig. 6A,B). Moreover, GSK-3β inhibition did not affect the aggregation of polyQ-expanded AR in PC12 cells (Fig. 6C). These findings suggest that the mechanism by which GSK-3β regulates AR nuclear export in prostate cancer cell lines does not reflect a general mechanism for AR nuclear export in other cellular contexts.

### Global disruption of nucleocytoplasmic transport is not observed in cell and animal models of SBMA

A number of studies have implicated disruptions in nucleocytoplasmic transport in the pathogenesis of neurodegenerative diseases such as ALS^73–77^ and HD^78,79^. Given that SBMA is both a neuromuscular disease (like ALS) as well as a polyQ repeat disorder (like HD), we sought to investigate whether cell and animal models of SBMA display evidence of global disruption in nucleocytoplasmic transport, and whether this could provide a mechanism for the impaired nuclear export of polyQ-expanded AR.

**Figure 7:**
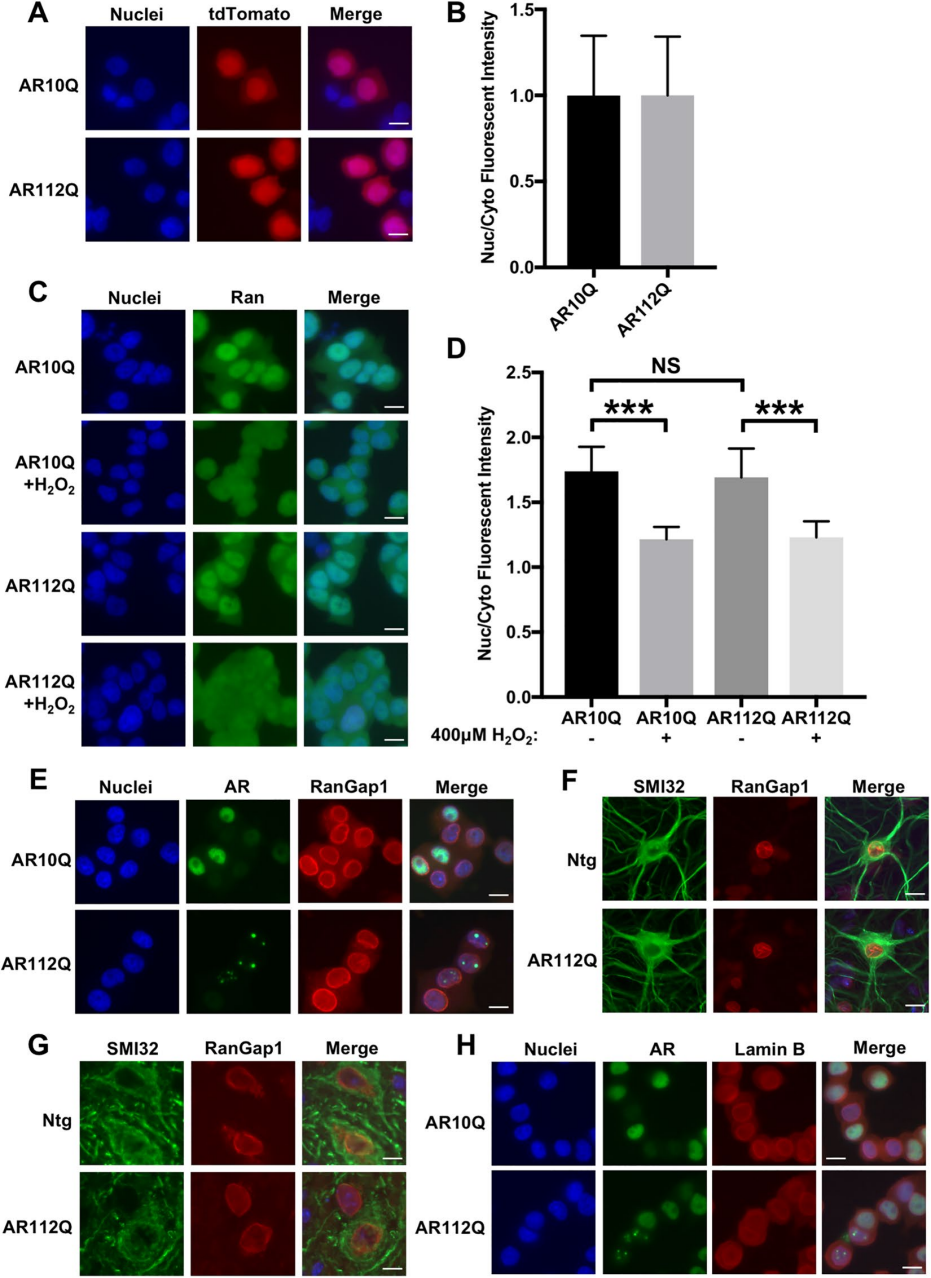
Global disruption of nucleocytoplasmic transport is not observed in cell and animal models of SBMA. (**A**) Immunofluorescence images of PC12 cells expressing AR10Q or AR112Q transiently transfected with NLS-tdTomato-NES and treated with 10 nM DHT for 48 hrs. (**B**) Quantification of (**A**) was performed by measuring the fluorescence intensity of NLS-tdTomato-NES in the nucleus versus the cytoplasm. 80–90 cells were analyzed per condition. (**C**) Ran staining in PC12 cells expressing AR10Q or AR112Q treated for 48 hrs with 10 nM DHT+/− 15 min with 400 μM H_2_O_2_. Short treatment of cells with H_2_O_2_ has been previously shown to disrupt the Ran gradient^96^. (**D**) Quantification of (**C**) was performed by measuring the fluorescence intensity of Ran in the nucleus versus the cytoplasm. 100 cells were analyzed per condition. Statistical significance was determined by one-way ANOVA with *post hoc* Tukey test. (**E**) RanGAP1 staining in PC12 cells expressing wildtype and polyQ-expanded AR treated with 10 nM DHT for 48 hrs. (**F**) RanGAP1 staining in primary motor neurons from AR112Q transgenic mice treated with DHT for 7 days. Motor neurons were identified by staining for neurofilament heavy chain by the SMI32 antibody and by morphology. (**G**) RanGAP1 staining in motor neurons from spinal cord sections of 17 month-old AR112Q transgenic male mice. Note that the merged images from (**F**) and (**G**) include Hoechst staining. (**H**) Lamin B staining in PC12 cells expressing AR10Q and AR112Q treated with 10 nM DHT for 48 hrs. ***p < 0.001. Experiments were repeated three times. Error bars = SD. Scale bars represent 10 μm.

To determine if nucleocytoplasmic transport is broadly disrupted in cells expressing mutant AR, we transiently transfected PC12 cells with tdTomato tagged with both a classical NLS and NES, which undergoes Ran-mediated nuclear import and export (a kind gift from J. Rothstein). Mislocalization of NLS-tdTomato-NES has been observed in primary cortical neurons expressing mutant huntingtin or TDP-43^77,78^, and deficient nuclear translocation of this protein was reported in neurons from C9orf72 ALS patient-derived induced pluripotent stem cells (iPSCs)^74^. In PC12 cells, expression of polyQ-expanded AR did not affect the nuclear/cytoplasmic distribution of NLS-tdTomato-NES (Fig. 7A,B). This suggests that Ran-mediated nuclear transport is not disrupted in PC12 cells expressing mutant AR. However, to further investigate this possibility, we assessed, using immunocytochemistry, the localization of specific nucleo/cytoplasmic transport proteins that are disrupted in cell and animal models of ALS and HD. Specifically, we examined AR112Q-expressing HEK293 cells and PC12 cells, as well as transgenic PrP-AR112Q primary motor neurons and spinal cord tissue from 17 month-old transgenic PrP-AR112Q mice for alterations in the localization of RanGAP1, a GTPase-activating protein normally located on the cytoplasmic filaments of the nuclear pore complex. RanGAP1 plays a key role in maintaining the nucleo/cytoplasmic gradient of Ran-GTP/GDP that drives active nuclear transport, and mislocalization of this protein occurs in numerous cell and animal models of ALS and HD, as well as in C9orf72 and HD patient brain tissue^74,76–79^. In cell and animal models of SBMA, however, we found no evidence of mislocalization of RanGAP1 or colocalization of RanGAP1 with AR intranuclear inclusions (Figs 7E–G and S2A).

Additionally, we found no disruptions in the Ran gradient in polyQ-expanded AR-expressing HEK293 or PC12 cells, further indicating that expression of polyQ-expanded AR does not disrupt Ran-mediated nucleo/cytoplasmic transport (Figs 7C,D and S2B). Finally, we examined the integrity of the nuclear membrane in PC12 cells by Lamin B staining and found no evidence of the structural abnormalities that have been observed in models of ALS and HD^73,77,79^ (Fig. 7H). Despite sharing common pathological features with these diseases, none of the SBMA models examined in this study displayed mislocalization of RanGAP1, disruptions in the Ran gradient, or altered nuclear membrane structure. Furthermore, none of these proteins were colocalized with AR intranuclear inclusion bodies, despite colocalizing with aggregates of mutant HD, poly(GA), and G_4_C_2_ mRNA. Thus, these experiments support a hypothesis in which an AR-specific process, rather than a global transport mechanism, leads to impaired nuclear export of polyQ-expanded AR.

### PolyQ-expanded AR exhibits reduced intranuclear mobility

**Figure 8:**
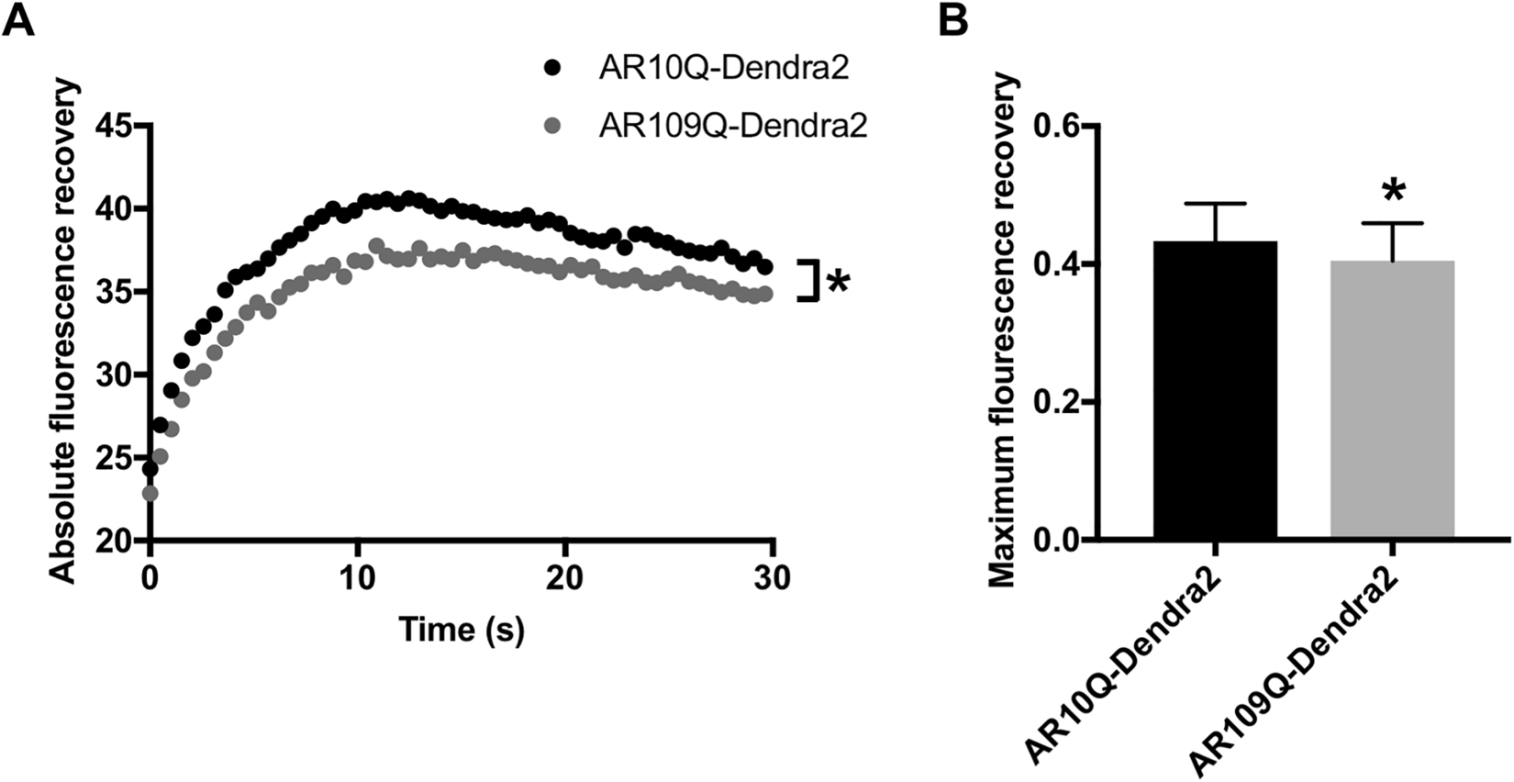
PolyQ-expanded AR exhibits reduced intranuclear mobility. (**A**) Averaged absolute FRAP curves of PC12 cells expressing AR10Q-Dendra2 or AR109Q-Dendra2 treated with 10 nM DHT for 24 hrs. 22 cells were analyzed per condition. Statistical significance was determined by two-way ANOVA. *p < 0.05. (**B**) Average maximum fluorescence recovery achieved by AR10Q-Dendra2 and AR109Q-Dendra2 post-photobleaching. Statistical significance was determined by one-tailed Student’s t-test. *p < 0.05. Experiment was repeated three times. Error bars = SD.

Previous studies have demonstrated that polyQ-expansion can decrease the intranuclear mobility of the protein within which it resides^44,80^. In order to determine if reduced intranuclear mobility of polyQ-expanded AR correlates with its increased nuclear retention, we performed fluorescence recovery after photobleaching (FRAP) analysis of cells stably expressing AR10Q-Dendra2 or AR109Q-Dendra2. In the absence of stimulation by UV light, proteins tagged with Dendra2 fluoresce in the green spectrum, allowing for visualization of live cells. This FRAP analysis revealed a modest, but statistically significant, decrease in the absolute fluorescence recovery rate of AR109Q-Dendra2 compared with AR10Q-Dendra2 over time. Additionally, we found a reduction in the maximum fluorescence recovery achieved by AR109Q-Dendra2 post-photobleaching (Fig. 8). It is possible that a slight disruption of intranuclear mobility could, along with other factors, contribute to the increased nuclear retention of mutant AR.

## Discussion

It has been widely reported that the ligand-dependent nuclear accumulation of mutant AR is required for SBMA pathogenesis^17,18,46^. This mechanistic insight into the toxicity of polyQ-expanded AR ultimately led to clinical studies that tested the effect of the androgen suppressors leuprorelin acetate and dutasteride on disease progression in SBMA patients^81–83^. Despite recent evidence that long-term treatment with leuprorelin acetate modestly slows disease progression^84^, overall the protective effects of androgen suppressors observed in SBMA animal models have not had a substantial impact on disease progression in symptomatic patients. The lack of an effective treatment for SBMA underscores the need for a better mechanistic understanding of aspects of mutant AR metabolism that can be targeted to ameliorate its toxic effects. To this end, the goal of this study was to determine if nuclear export, a poorly understood step in the metabolism of AR, plays a role in SBMA pathogenesis and whether reducing nuclear AR by enhancing its export can reduce its aggregation and toxicity.

The results of our study demonstrate (1) that the nuclear export of polyQ-expanded AR is impaired, (2) that enhancing the nuclear export of polyQ-expanded AR with an exogenous NES decreases its aggregation and toxicity by promoting its proteasomal degradation, (3) that polyQ-expanded AR exhibits reduced phosphorylation at S650 and that blocking S650 phosphorylation further exacerbates its nuclear export deficiency, and (4) that global disruption of nucleo/cytoplasmic transport is not a common feature of SBMA models.

Deficient nuclear export of mutant AR may contribute to its toxicity, as persistence in the nucleus could lead to aberrant protein-protein interactions, thereby disrupting normal cellular processes. Indeed, lack of nuclear export of polyQ-expanded ataxin-1^45^ correlates with altered protein-protein interactions that contribute to its toxicity by both gain-of-function and loss-of-function mechanisms^85^. An important question for future studies is whether AR nuclear export becomes acutely impaired at a polyQ tract length of 38, the threshold for fully penetrant disease in SBMA patients^1^. It also remains to be determined if longer polyQ tract lengths correlate with increasing impairment of AR nuclear export.

In order to understand why the nuclear export of mutant AR is impaired, we turned to previously published insights into the mechanism by which wildtype AR undergoes nuclear export. This led to an investigation of the status of S650 phosphorylation of polyQ-expanded AR, as phosphorylation at this residue had been shown to regulate the nuclear export and nuclear accumulation of wildtype AR^34,35^. Intriguingly, polyQ-expanded AR exhibits substantially reduced pS650 compared with wildtype AR; moreover, genetic mutation to prevent phosphorylation of S650 further disrupted the nuclear export of polyQ-expanded AR. These results suggest that the reduced phosphorylation of S650 in polyQ-expanded AR may contribute to its deficient nuclear export.

Phosphorylation of the AR is known to regulate numerous aspects of AR function by modulating its interactions with coactivators^86^. Specifically, expression of the AR coactivator SNURF (small nuclear ring finger protein/ring finger protein 4) has been shown to slow AR nuclear export and increase AR association with the nuclear matrix^28^. AR phosphorylation at S650 may function similarly, facilitating nuclear export by altering the interactions between AR and its binding partners, perhaps including exportins. Furthermore, changes in the AR interactome that occur upon S650 phosphorylation may also contribute to the process of AR aggregation.

While reduced pS650 represents one mechanism by which the nuclear export of polyQ-expanded AR is impaired, the regulation of AR nuclear export likely depends on other signals that remain to be identified. Our data indicate that phosphorylation of S650 is not absolutely necessary for AR nuclear export in PC12 cells (as AR S650A 109Q still undergoes nuclear export), nor is a phospho-mimic S650D mutation sufficient to rescue the impaired nuclear export of polyQ-expanded AR. Nevertheless, elucidating the precise mechanism by which pS650 regulates AR nuclear export will be important for developing a better understanding of this process.

The relationship between the role of S650 in regulating AR nuclear export and its regulation of other aspects of AR metabolism could offer new insights into how these pathways converge in SBMA. In the present study, we found that the S650D mutation increased the frequency of cells with intranuclear inclusions of polyQ-expanded AR, demonstrating a role for this residue in AR aggregation. A pressing question is whether facilitation of the slow, CRM1-independent nuclear export mechanism for AR actually contributes to its misfolding and aggregation, as suggested by this observation; resolving this question will be the subject of future investigation. Additionally, *in vitro* studies offer evidence for the role of S650 on AR transcriptional activity, finding that a phospho-null S650A mutation decreases AR transcriptional activity to varying degrees^34,87–89^. In humans, an S650G mutation has been associated with partial androgen insensitivity syndrome^89,90^, and alterations in AR phosphorylation at S650 have been reported in breast cancer tumors^91^. Taken together, S650 appears to regulate several AR functions relevant to SBMA, making it an intriguing subject for further study.

Another previously reported mechanism of AR nuclear export that we examined in our cell models is the CRM1-dependent nuclear export of AR that occurs in several prostate cancer cell lines upon inhibition of GSK-3β^29,30,65^. We were particularly intrigued by the possibility of pharmacologically directing AR export through CRM1, as this would mirror the mechanism by which the exogenous NES tag we utilized in our experiments directs nuclear export^56^. In our cell models, however, GSK-3β inhibition had no effect on the subcellular localization of AR. This result, along with our finding that S650 phosphorylation did not affect wildtype AR nuclear export in PC12 cells, suggests that previously reported mechanisms of AR export may not be strong regulators of polyQ-expanded AR nuclear export. This highlights the need for further investigation into the molecular mechanisms underlying the deficient nuclear export of polyQ-expanded AR.

Despite a growing body of evidence that cell-wide disruption of nucleo/cytoplasmic transport is a common pathological hallmark of ALS, HD, and other neurodegenerative disorders, we found no evidence for such disruptions in the SBMA models examined in this study. While we cannot rule out the possibility that nucleo/cytoplasmic transport and/or nuclear membrane structure is perturbed in SBMA patient tissue, in the SBMA models investigated here, which display numerous aspects of AR aggregation and toxicity characteristic of the disease, no global disruption was observed. This finding indicates that, in these models, a general disruption of nucleo/cytoplasmic transport is not present, and thus not responsible for AR nuclear retention and toxicity. Notably, even severely symptomatic transgenic PrP-AR112Q mice exhibit no mislocalization of RanGap1, suggesting that the toxicity caused by expression of polyQ-expanded AR is not mediated by defects in nuclear trafficking. Indeed, our observation that nucleo/cytoplasmic transport is not disrupted in SBMA models agrees with a recent study in which aggregation-prone artificial β-sheet proteins targeted to the cytoplasm, but not to the nucleus, interfered with nucleo/cytoplasmic transport machinery of transfected HEK293T cells^92^. While cytoplasmic AR aggregates have been reported in SBMA patient tissue (particularly in sensory neurons of dorsal root ganglia)^93,94^, nuclear aggregates are more frequently observed and are considered to be a hallmark of disease pathology. Our data support the theory that nuclear aggregates do not disrupt nucleo/cytoplasmic transport machinery, highlighting an important difference between the pathology of SBMA and related neurodegenerative disorders.

This study offers the first evidence that the nuclear export of polyQ-expanded AR is impaired, highlighting a novel aspect of AR metabolism that may contribute to the pathogenesis of SBMA. Furthermore, our data support a model in which the mutant AR is rapidly degraded by the proteasome once exported from the nucleus, thereby decreasing its stability, aggregation, and toxicity. Although additional work will be required to fully elucidate the mechanism underlying deficient nuclear export of polyQ-expanded AR, our results provide a new direction for investigations into therapeutic manipulation of the mutant, polyQ-expanded AR in SBMA.

## Materials and Methods

### Cell Culture and Reagents

PC12 cells were grown in Dulbecco’s modified Eagle’s medium (DMEM) supplemented with 10% heat-inactivated horse serum, 5% heat-inactivated fetal bovine serum, 2mM L-glutamine, 100 units/mL penicillin/streptomycin, 200 μg/mL hygromycin, and 100 μg/mL G418 Sulfate. HEK293 cells were maintained in DMEM supplemented with 10% heat-inactivated fetal bovine serum, 2mM L-glutamine, 100 units/mL penicillin/streptomycin, 15 μg/mL blasticidin S, and 100 μg/mL hygromycin. NIH/3T3 cells were maintained in DMEM supplemented with 10% heat-inactivated fetal bovine serum, 2mM L-glutamine, and 100 units/mL penicillin/streptomycin. All cells were maintained at 37 °C, 10% CO_2_. Experiments were performed utilizing charcoal-stripped serum. For the analysis of AR degradation, cells were treated with 20 μM MG132 (to inhibit the proteasome) or 5 mM 3-MA (to inhibit autophagy) for 24 hrs. Cells were lysed and analyzed by western blot as described below.

### Heterokaryon Shuttling Assay

The heterokaryon shuttling assay for analysis of AR nuclear export was carried out using modifications of the protocol of Black *et al*., 2001. Stable Tet-On PC12 cells expressing AR10Q or AR112Q were treated with 500 ng/mL doxycycline for 24 hrs to induce AR expression. Separately, NIH/3T3 mouse fibroblast cells were plated in complete media. Prior to fusion, actively growing NIH/3T3 cells were stained with 1 μM CellTracker^TM^ Orange CMTMR Dye in Optimem for 45 min, then incubated for an additional 60 min in complete media. Concurrently, PC12 cells were washed of doxycycline and treated for 2 hrs with 10 nM DHT (to induce nuclear localization of the AR) and 10 μg/mL cycloheximide (to inhibit protein synthesis).

PC12 cells and stained NIH/3T3 cells were trypsinized, mixed in Optimem at a 1:1 ratio, and then pelleted. The cells were fused in suspension with 100 μL 50% PEG-1500 (w/v) added slowly over 2 min. The cell/PEG-1500 mixture was then diluted with 5 mL Optimem, added slowly during an additional 3 min. The fused cells were pelleted and re-plated onto poly-D-lysine coated coverslips in the presence of 10 nM DHT and 10 μg/mL cycloheximide for 4 hrs prior to fixation and immunostaining.

### Immunostaining

Immunostaining was carried out as described by Montie *et al*., 2009 following 20 min fixation in 4% paraformaldehyde. Spinal cord tissue was embedded in OCT and cryosectioned (7μm) prior to fixation. All animal procedures were performed following the guidelines of the Office of Laboratory Animal Welfare and with the approval of the Thomas Jefferson University Institutional Animal Care and Use Committee.

Antibodies used include: AR (H280, Santa Cruz Biotechnology), AR (AR-318-CE, Leica Biosystems), AR pS650 (ab47563, Abcam), RanGAP1 (H180, Santa Cruz Biotechnology), Ran (610340, BD Transduction Laboratories), Lamin B1 (ab16048, Abcam), HDAC6 (D21B10, Cell Signaling Technology), HSP70 (SMC-100, StressMarq), neurofilament-heavy chain (SMI32; 801701 Sternberger Monoclonals), and 3B5H10 (a kind gift from Dr. Steve Finkbeiner, Gladstone Institute of Neurological Disease and UCSF). Immunostained cells were visualized using a Leica DMR Fluorescence microscope (Leica Microsystems GmbH, Wetzlar, Germany) and imaged using iVision Mac® or ProgRes® software.

In PC12 cells, the percentage of inclusions was determined by counting at least 300 cells in triplicate wells. Experiments were repeated at least three times.

### Western blot analysis

Protein extracts were prepared by lysing cells in Triton-DOC lysis buffer (1% sodium deoxycholate, 0.5% Triton X-100 in PBS with protease inhibitors). All lysates were sonicated at least three times for 10 s using a Branson cup sonifier. Protein concentration was determined by DC protein assay (Bio-Rad). Lysates were electrophoresed by SDS-PAGE and transferred to 0.45 μm PVDF membrane (Immobilon-P). Blots were incubated with primary antibodies diluted in 5% nonfat milk in TBST (TRIS-buffered saline, 0.1% Tween-20) for 60 min at room temperature or overnight at 4 °C and with HRP-conjugated secondary antibodies (mouse: AP308P, rabbit: AP307B; Millipore) for 60 min at room temperature. Detection was performed using ECL substrate (Thermo Fisher) with analysis on a ChemiDoc MP (Bio-Rad).

Antibodies used include: AR (H280, Santa Cruz Biotechnology), AR pS650 (ab47563, Abcam), α-tubulin (21445, Cell Signaling Technology), β-catenin (8480 T, Cell Signaling Technology), Lamin B1 (ab16048, Abcam), p62 (ab56416, Abcam), and GAPDH (10R-G109A, Fitzgerald).

### PC12 Cell Toxicity Assay

Stable Tet-On PC12 cells expressing AR112Q or NES-AR108Q were treated with 500 ng/mL doxycycline (to express equivalent levels of AR) in the presence and absence of 10 nM DHT for 12 days. The percentage of dead cells was determined by counting the percentage of Trypan blue-positive cells; samples were blinded prior to counting. Three independent experiments were performed in triplicate with at least two hundred cells counted per condition.

### SDS-agarose gel electrophoresis (SDS-AGE)

PC12 cells were lysed in Triton-DOC lysis buffer (1% sodium deoxycholate, 0.5% Triton X-100 in PBS with protease inhibitors) and diluted 1:1 in non-reducing Laemmli sample buffer. Samples were boiled for 5 minutes and electrophoresed through a 1% agarose gel containing 0.1% SDS in 375 mM Tris-HCL, pH 8.8, for approximately 18 hrs at 4 °C. Lysates were then transferred to a 0.45 μm PVDF membrane at 200 mA for 3 hrs using a semi-dry transfer apparatus (Owl HEP Semidry Electroblotting Systems, Thermoscientific). A 4 kg weight was placed on top of the transfer apparatus to accommodate thinning of the agarose gel during transfer. Western analysis was performed using anti-AR (H280, Santa Cruz Biotechnology) or 3B5H10 (a kind gift from Dr. Steve Finkbeiner, Gladstone Institute of Neurological Disease and UCSF) antibodies.

### Protein Turnover Assay

AR expression was induced with doxycycline in PC12 cell lines expressing AR112Q or NES-AR108Q for 24 hrs. Cells were then washed with PBS to remove media containing doxycycline and treated with media containing 10 nM DHT for 2 hrs. Subsequently, cells were treated with media containing 10 nM DHT and 10μg/mL cycloheximide for 10 hrs. Cells were harvested at the given time points after the final media replacement and analyzed by Western blot, as described above.

### Creation of clonal PC12 cell lines

The NES-AR plasmid was constructed by excision of pCMV NES-AR (N-terminal fragment) (a kind gift from Andrew Lieberman, University of Michigan) with SnaBI/EagI and ligation into a preexisting pTRE AR112Q plasmid by sequential digests with NheI (blunted with DNA polymerase I, large (Klenow) fragment) and EagI. The NES utilized in this study (ATGTTAGCCTTGAAATTAGCAGGATTAGACATC) was originally identified in cAMP-dependent protein kinase inhibitor alpha^56^.

AR-Dendra2 plasmids were constructed by mutation of the AR STOP codon to an AgeI restriction enzyme site by PCR, excision of the AR C-terminus from this PCR product by BstBI/AgeI digestion, and ligation into a pGWI-Dendra2 plasmid (kind gift of Dr. Steven Finkbeiner, Gladstone Institute of Neurological Disease and UCSF). C-terminally tagged AR-Dendra2 was then excised with BstBI/XbaI and ligated into preexisting pTRE AR10Q and pTRE AR112Q plasmids.

Mutation of AR Serine 650 to alanine or aspartate was performed by site-directed mutagenesis (QuikChange II, Stratagene) of the pTRE AR10Q and pTRE AR112Q plasmids. The AR coding region was then sequenced to verify the absence of secondary mutations.

Stable transfection of Tet-On PC12 cells with these AR constructs and a plasmid conferring hygromycin resistance (pTK-hygromycin) in a 1:4 molar ratio was then performed using lipofectamine 2000 according to the manufacturer’s instructions. Stable transformants were selected with 200 μg/mL hygromycin. Single colonies were isolated and screened for AR protein expression by induction with 1 μg/mL doxycycline and western blotting using an anti-AR antibody (H280, Santa Cruz Biotechnology). Genomic DNA was extracted from positive clones, and the CAG repeat length was verified by PCR and sequencing analysis. The concentration of doxycycline required to induce equivalent levels of AR protein between cell lines was determined and used for all experimentation.

### Immunoprecipitation

PC12 cells were pelleted in PBS and lysed in NP-40 buffer (20 mM HEPES-NaOH pH 7.9, 100 mM NaCl, 2 mM MgCl2, 0.1% NP-40) supplemented with protease and phosphatase inhibitors. Lysates were incubated on ice for 30 min and cleared by centrifugation. Protein concentration was determined using a DC protein assay (Bio-Rad) and samples were diluted to 1 mg/mL for the IP reaction with 10μg reserved for the input control. 0.5 mg of lysates was incubated with 1μg of AR antibody (BD Biosciences) or mouse IgG, crosslinked to Dynabeads M-270 Epoxy (Invitrogen) overnight at 4 °C with rotation. Immunoprecipitated protein was washed 3–4 times with lysis buffer and eluted with Laemmle buffer for western analysis.

### Proximity Ligation Assay

PC12 cells were plated on poly-D-lysine coated glass coverslips and treated with 500 ng/mL doxycycline and 10 nM DHT for 24 hrs. Duolink® PLA Fluorescence protocol was performed per manufacturer’s instructions (DUO92008, Sigma-Aldrich) with primary antibodies against total AR (AR-318-CE, Leica Biosystems) and AR pS650 (ab47563, Abcam). Images were acquired with the same exposure time.

### Dissociated Spinal Cord Cultures

Dissociated spinal cord cultures were established as previously described^17,95^. Briefly, spinal cords were dissected from embryonic day 13.5 PrP-AR112Q transgenic mice, dissociated with trypsin, and plated on poly-D-lysine coated coverslips in 24-well plates. Cultures were grown in glia-conditioned media containing minimal essential medium, 3% charcoal-stripped horse serum, 35 mM NaHCO_3_, 0.5% dextrose, 1% N3, and 10 nM 2.5 S nerve growth factor for 3 weeks. Cultures were then treated with either ethanol or 10μM DHT for 7 days. Motor neurons were identified by the presence of neurofilament-heavy chain (SMI32 immunoreactivity) and morphology.

### Fluorescence Recovery After Photobleaching

PC12 cells stably expressing AR10Q-Dendra2 or AR109Q-Dendra2 were plated on poly-D lysine-coated glass bottom dishes (MatTek Corp). AR expression was induced with 500 ng/mL doxycycline and cells were treated with 10 nM DHT for 24 hrs. Prior to imaging, media was replaced with Opti-MEM I minus phenol red (Gibco), supplemented with 500 ng/mL doxycycline and 10 nM DHT. Live cell imaging was performed on a Nikon A1R microscope with cells maintained at 37 °C and 5% CO_2_. Cells of approximately the same starting intensity were selected for photobleaching in a designated region of interest (ROI) within the cell nucleus, excluding the nucleolus. Photobleaching was performed using the 490 nm later at 10% power for 4 iterations. Fluorescent recovery within the bleached zone was measured every 0.5 sec for 30 sec, using the 490 nm later at 0.7% power. Absolute fluorescent recovery was determined by the percent of fluorescent recovery relative to the starting (pre-bleach) fluorescent intensity of the ROI. Maximum recovery represents the greatest percent of fluorescent recovery within an ROI independent of time.

### Statistical analysis

For pairwise comparisons, statistical significance was determined by two-tailed Student’s t-test unless otherwise noted in figure legends. For multiple comparisons, statistical significance was determined by one-way analysis of variance (ANOVA) with post-hoc Tukey test. For comparison of distributions, statistical significance was determined by the Kolmogorov-Smirnov test. The significance level (α) was set at 0.05 for all experiments. Data were compiled and analyzed using Microsoft Excel or Prism 7 (GraphPad).

## Electronic supplementary material

Supplementary Document 
