# Supplementary material for: Impaired Nuclear Export of Polyglutamine-Expanded Androgen Receptor in Spinal and Bulbar Muscular Atrophy
Source: Sci Rep. 2019 Jan 15;9:119. doi: 10.1038/s41598-018-36784-4 (PMC6333819; doi:10.1038/s41598-018-36784-4)
Supplement: Supplementary file 1 — Supplementary Document [file 41598_2018_36784_MOESM1_ESM.pdf]

SUPPLEMENTARY INFORMATION

IMPAIRED NUCLEAR EXPORT OF POLYGLUTAMINE-EXPANDED ANDROGEN RECEPTOR IN SPINAL AND BULBAR MUSCULAR ATROPHY

Frederick J. Arnold, Anna Pluciennik, and Diane E. Merry
Department of Biochemistry and Molecular Biology, Thomas Jefferson University, Philadelphia, PA 19107

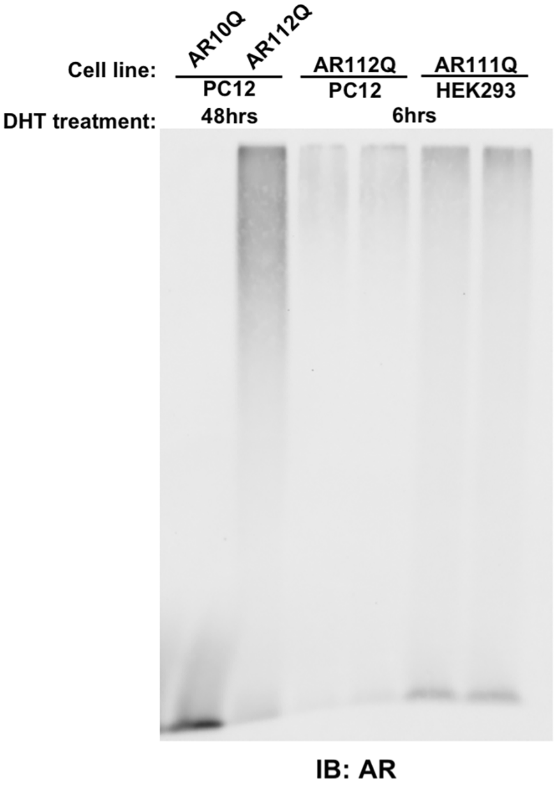

Figure S1. SDS-agarose gel electrophoresis (SDS-AGE) analysis of AR aggregation species following 6 hrs DHT treatment.

Western analysis of PC12 and HEK293 cell lysates resolved by SDS-AGE following treatment with 10nM DHT and 10µg/mL cycloheximide for 6 hrs. Note: this reflects the treatment of cells utilized for the heterokaryon shuttling assay, in which cells were treated with 10nM DHT and 10µg/mL cycloheximide for 2 hrs pre-fusion and for 4 hrs post-fusion.

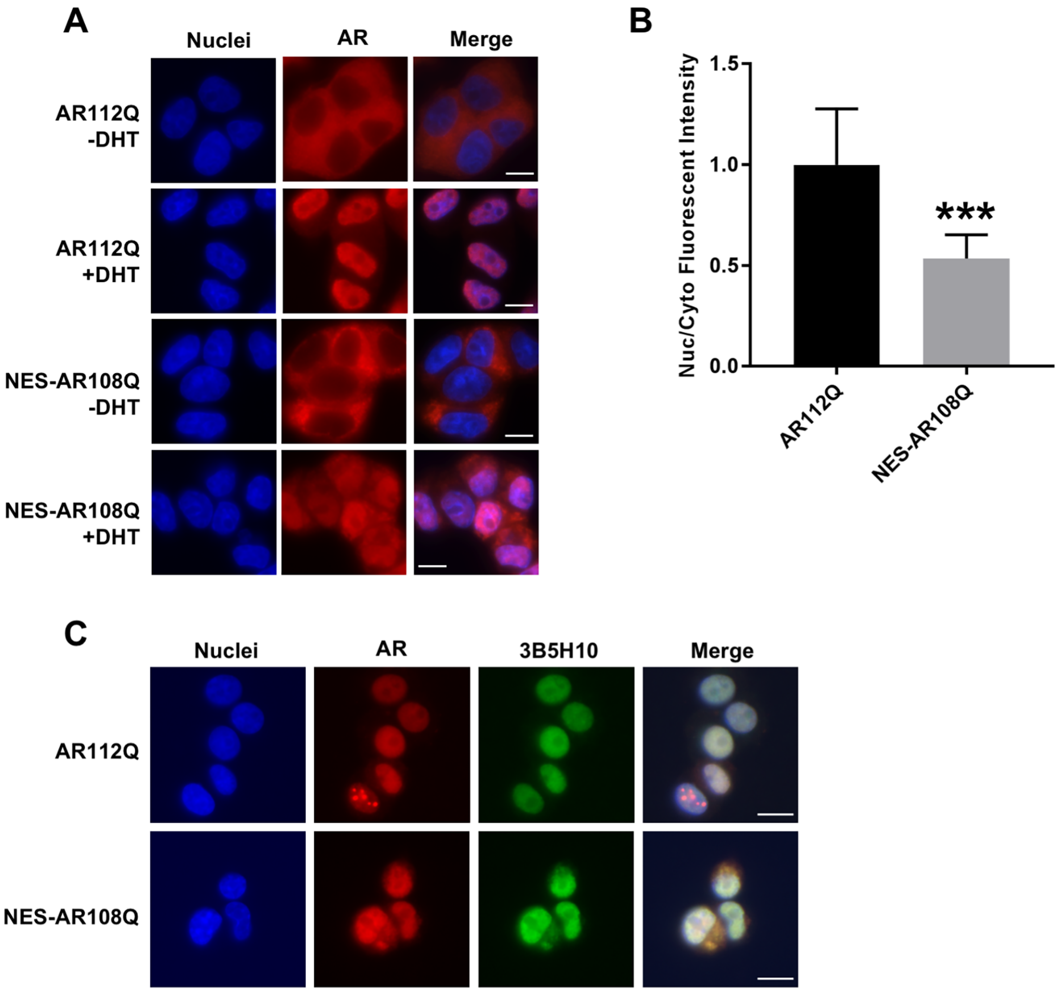

Figure S2. Supplementary experiments pertaining to Figure 2.

(**A**) Immunofluorescence images of PC12 cells expressing AR112Q or NES-AR108Q following 24 hrs treatment with 10nM DHT or ethanol (vehicle). (**B**) Quantification of the nuclear/cytoplasmic distribution of AR112Q and NES-AR108Q was performed by measuring the fluorescence intensity of AR in the nucleus versus the cytoplasm. 100 cells were analyzed per condition. Statistical significance was determined by Student's t test. ***$p < 0.001$. (**C**) Immunofluorescence images of PC12 cells expressing AR112Q or NES-AR108Q following 24 hrs treatment with 10nM DHT. Note: the 3B5H10 antibody detects NES-AR108Q in both the cytoplasm and the nucleus. Scale bars represent 10μm.

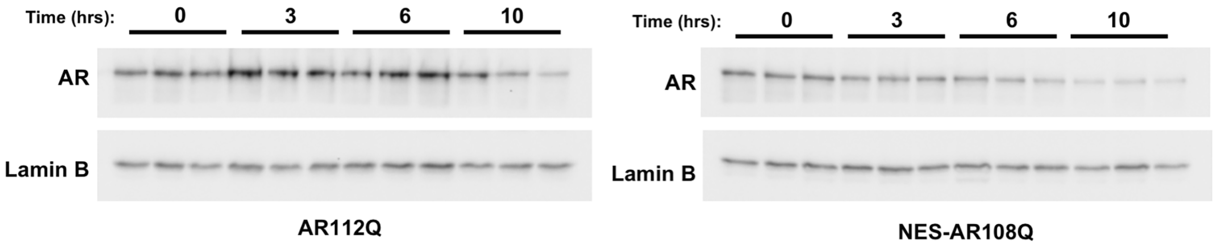

Figure S3. Supplementary data pertaining to Figure 3F. Determination of AR half-life. PC12 cells expressing AR112Q or NES-AR108Q were treated with DOX for 24 hrs. Following DOX washout, cells were treated with 10nM DHT for 2 hrs, then with 10nM DHT and 10μg/mL cycloheximide for the indicated times.

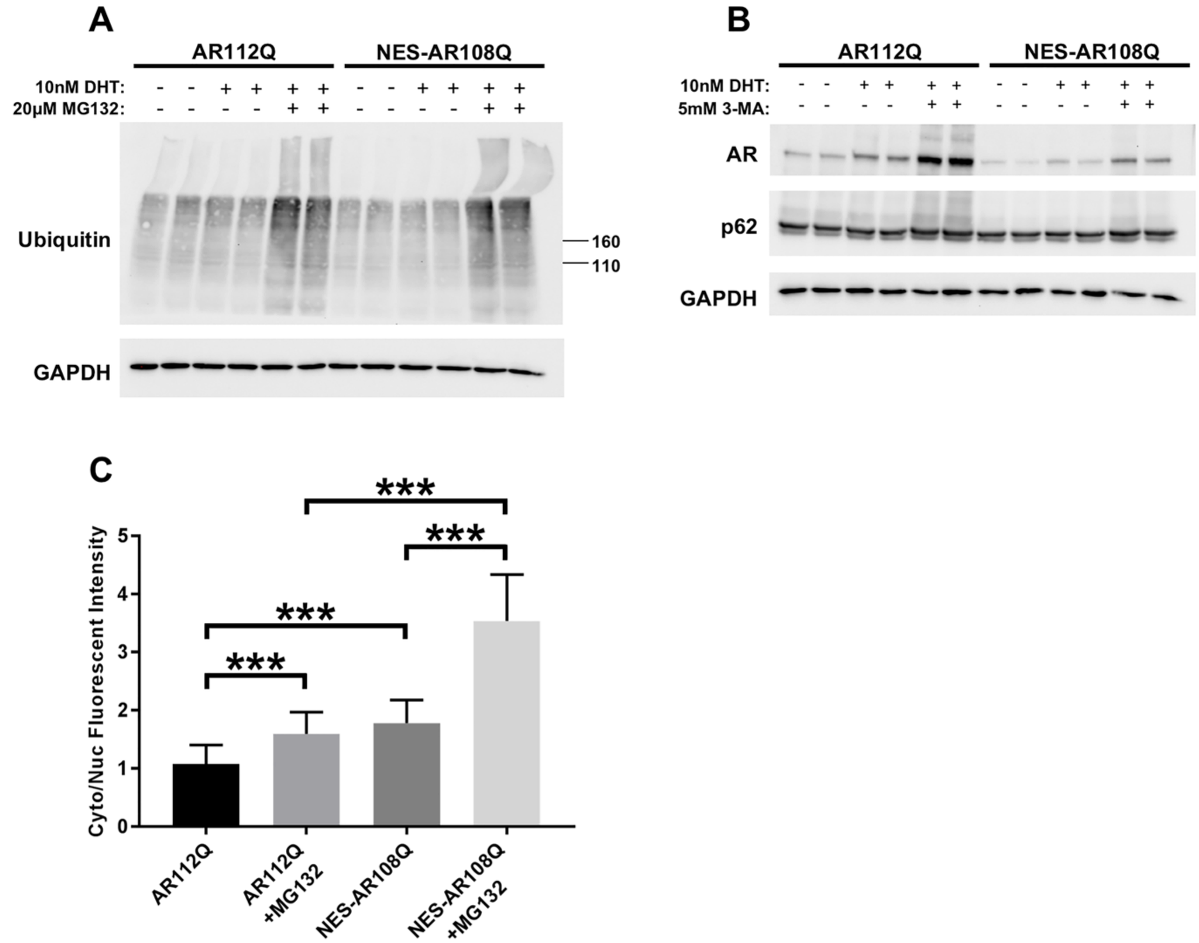

Figure S4. Supplementary experiments pertaining to Figure 3A-E.

(**A**) PC12 cells expressing AR112Q or NES-AR108Q were treated with DOX for 24 hrs. Following DOX washout, cells were treated for an additional 24 hrs with 10nM DHT or ethanol (vehicle) and 20μM MG132 or DMSO (vehicle) and analyzed by Western blot. Note: treatment of PC12 cells with MG132 causes a substantial increase in ubiquitinated protein. (**B**) PC12 cells expressing AR112Q or NES-AR108Q were treated with DOX for 24 hrs. Following DOX washout, cells were treated for an additional 24 hrs with 10nM DHT or ethanol (vehicle) and 5mM 3-MA or H_2O (vehicle) and analyzed by Western blot. Note: treatment of PC12 cells with 3-MA leads to an increase in p62 protein levels, as well as an increase in AR stability as previously reported [1]. (**C**) Quantification of the fluorescence intensity of AR112Q and NES-AR108Q in the cytoplasm versus the nucleus following 24 hrs treatment with 10nM DHT and 10μM MG132 or DMSO (vehicle). 100 cells were analyzed per condition. Statistical significance was determined by one-way ANOVA with *post hoc* Tukey test. ***p < 0.001.

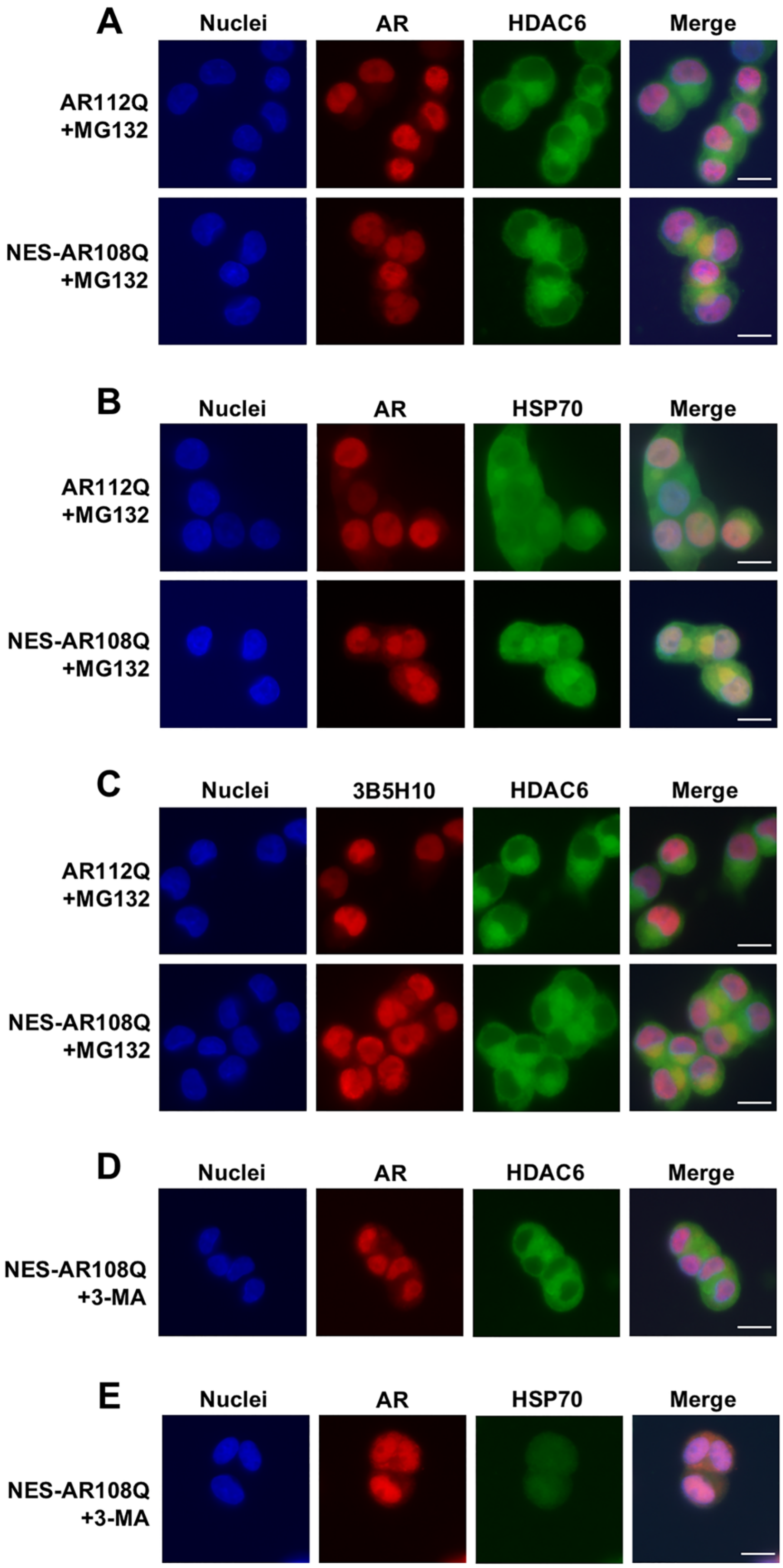
A
Nuclei
AR
HDAC6
Merge
AR112Q
+MG132
NES-AR108Q
+MG132
B
Nuclei
AR
HSP70
Merge
AR112Q
+MG132
NES-AR108Q
+MG132
C
Nuclei
3B5H10
HDAC6
Merge
AR112Q
+MG132
NES-AR108Q
+MG132
D
Nuclei
AR
HDAC6
Merge
NES-AR108Q
+3-MA
E
Nuclei
AR
HSP70
Merge
NES-AR108Q
+3-MA

Figure S5. Supplementary data pertaining to Figure 3E. Evaluation of AR-containing aggresomes.

(**A**-**C**) Immunofluorescence images of PC12 cells expressing AR112Q or NES-AR108Q treated for 24 hrs with 10nM DHT and 10μM MG132 or DMSO (vehicle). (**D**, **E**) Immunofluorescence images of PC12 cells expressing AR112Q or NES-AR108Q treated for 24 hrs with 10nM DHT and 5mM 3-MA or H_2O (vehicle). Note: treatment of PC12 cells with 3-MA does not lead to the formation of AR-containing aggresomes. Scale bars represent 10μm.

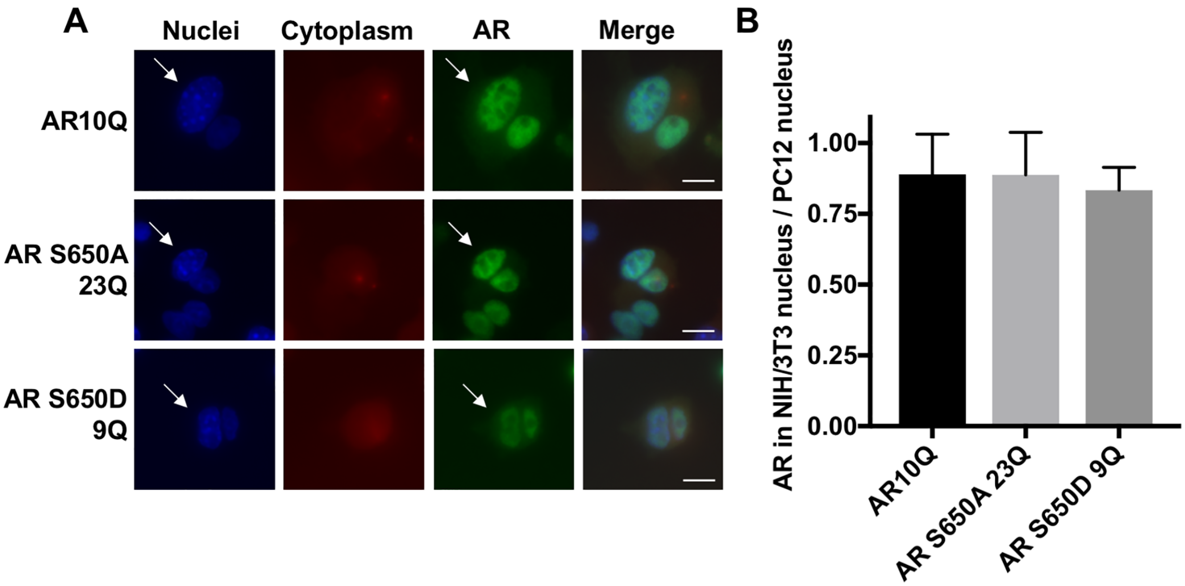

Figure S6. Mutation of Serine 650 does not affect nuclear export of wildtype AR in PC12 cells.

(**A**) Heterokaryon analysis of PC12 cells expressing AR10Q, AR S650A 23Q, and AR S650D 9Q. The acceptor (NIH/3T3) nucleus is indicated by white arrows. Scale bars represent 10μm. (**B**) Quantification of the fluorescence intensity of AR in the acceptor nucleus relative to the donor nucleus. 20-25 heterokaryons were analyzed per condition. Experiment was repeated three times. Error bars = SD.

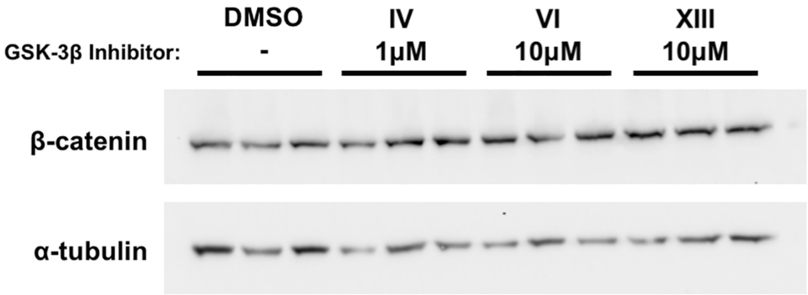

Figure S7. Supplementary data pertaining to Figure 6D. GSK-3β inhibitors stabilize β-catenin.

Western analysis of HEK293 cells expressing AR10Q treated with GSK-3 inhibitor IV, GSK-3β inhibitor VI, or DMSO (vehicle) for 48 hrs or with GSK-3 inhibitor XIII for 24 hrs (shortened treatment due to toxicity of the compound).

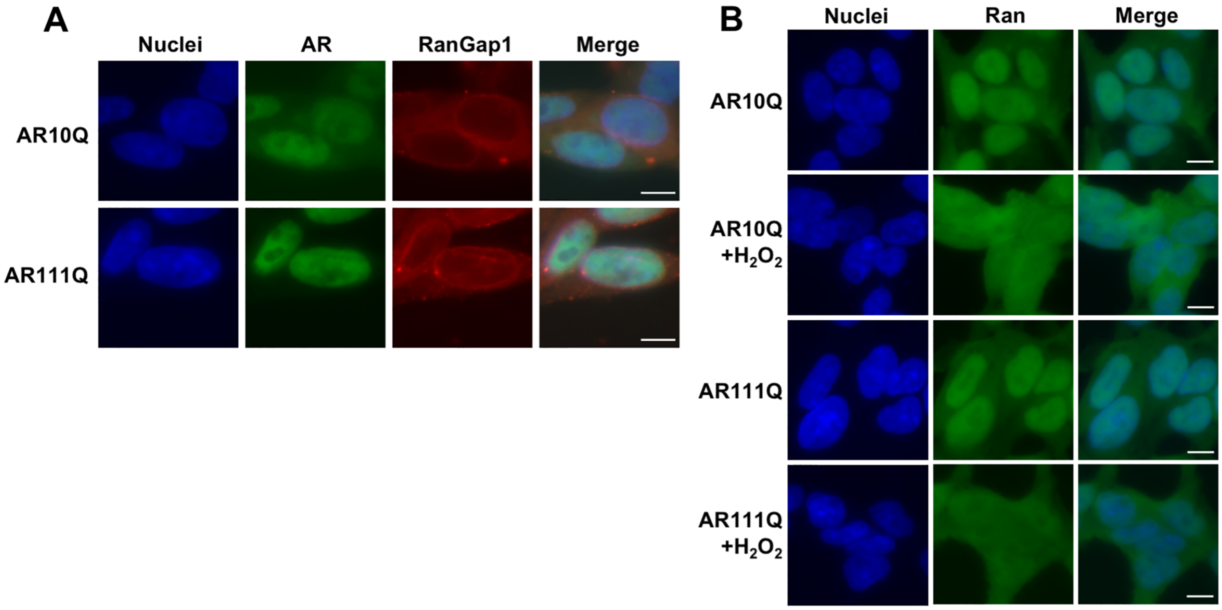

Figure S8. Global disruption of nucleocytoplasmic transport is not observed in cell and animal models of SBMA.

(**A**) RanGAP1 staining in HEK293 cells expressing wildtype and polyQ-expanded AR treated with 10nM DHT for 48 hrs. (**B**) Ran staining in HEK293 cells expressing AR10Q or AR111Q treated for 48 hrs with 10nM DHT +/- 15min with 400μM H_2O_2. Short treatment of cells with H_2O_2 has been previously shown to disrupt the Ran gradient [2]. Experiments were repeated three times. Scale bars represent 10μm.

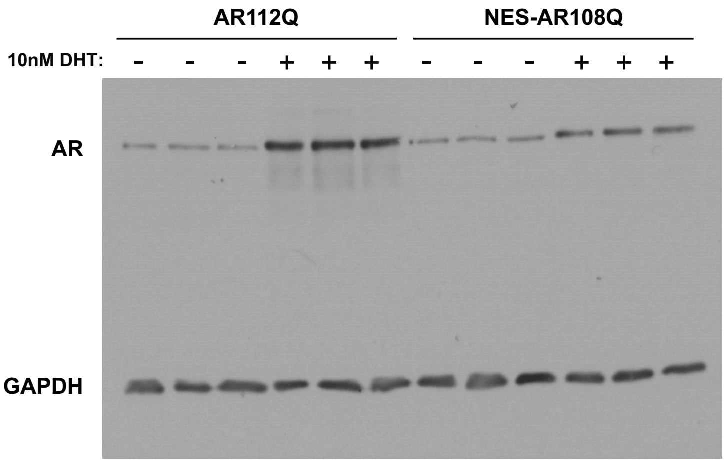

Figure S9. Full-length western blot corresponding to Figure 2A.

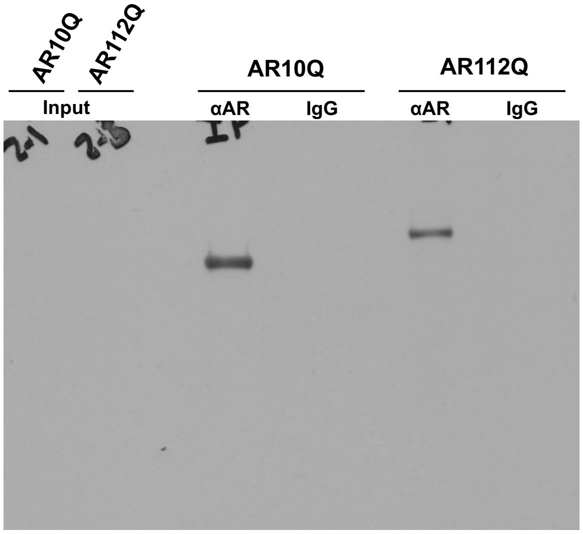

IB: AR pS650

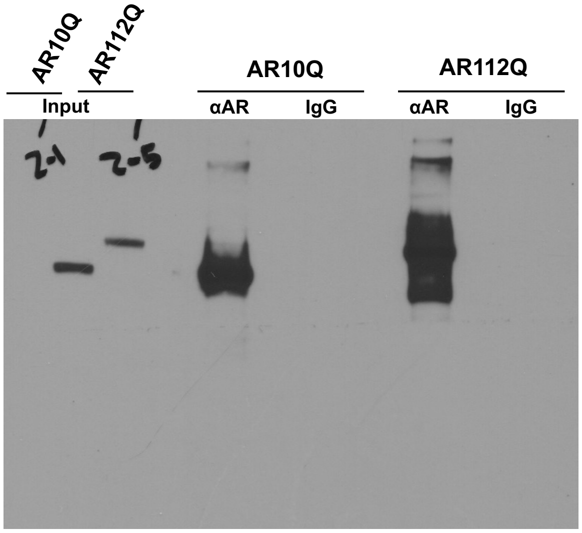

IB: Total AR
Darker exposure

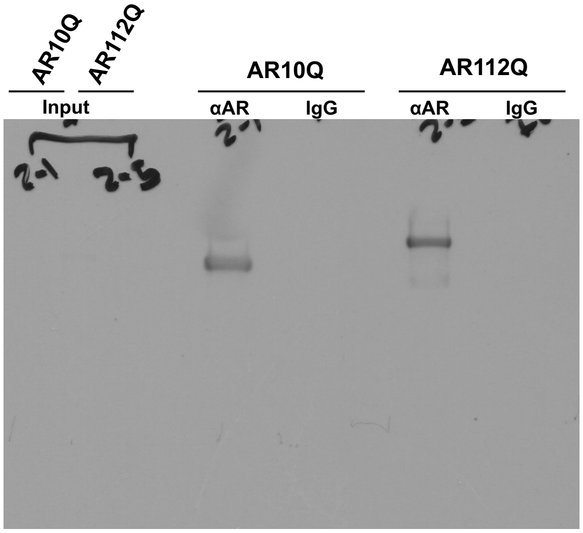

IB: Total AR
Lighter exposure

Figure S10. Full-length western blots corresponding to Figure 4A.

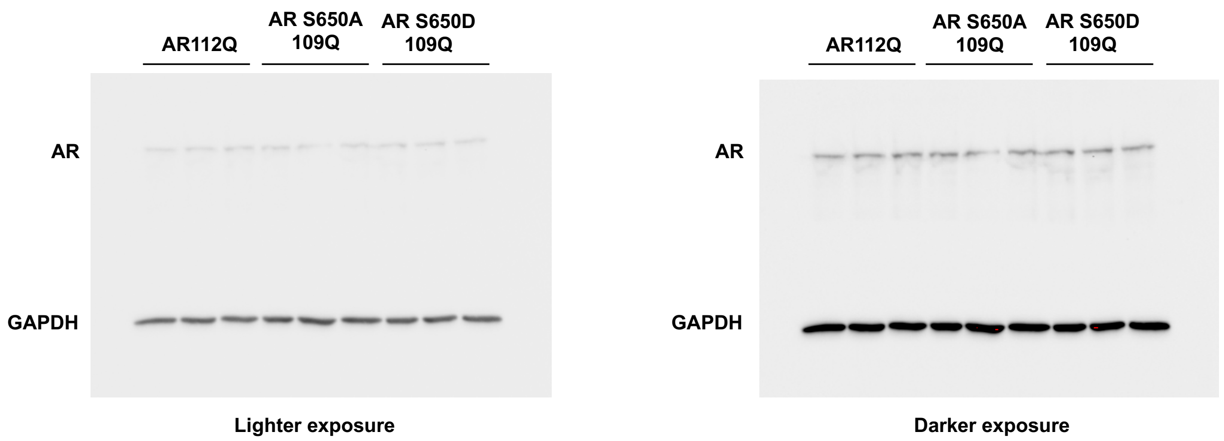

Figure S11. Full-length western blots corresponding to Figure 5A.

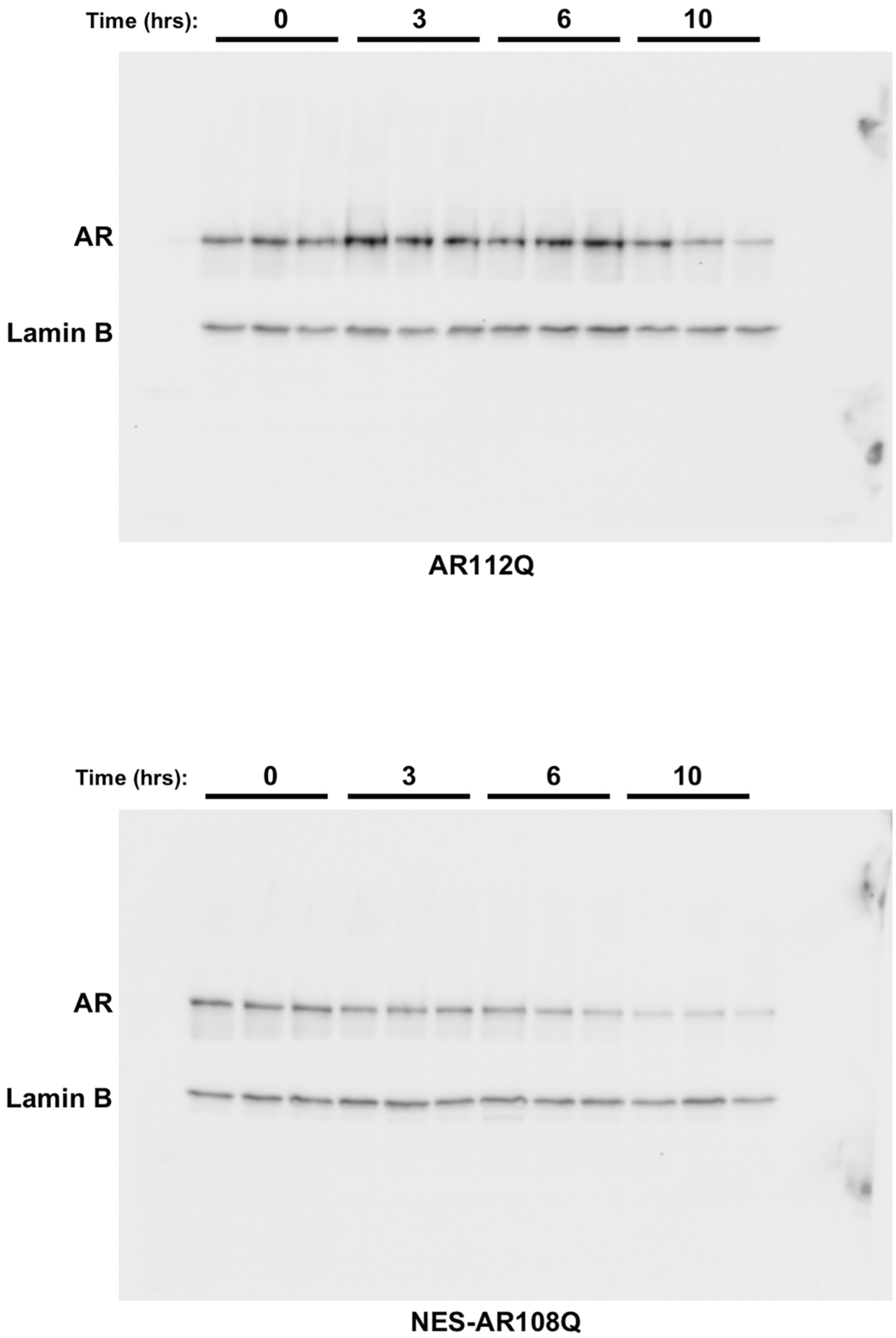

Figure S12. Full-length western blots corresponding to Figure S3.

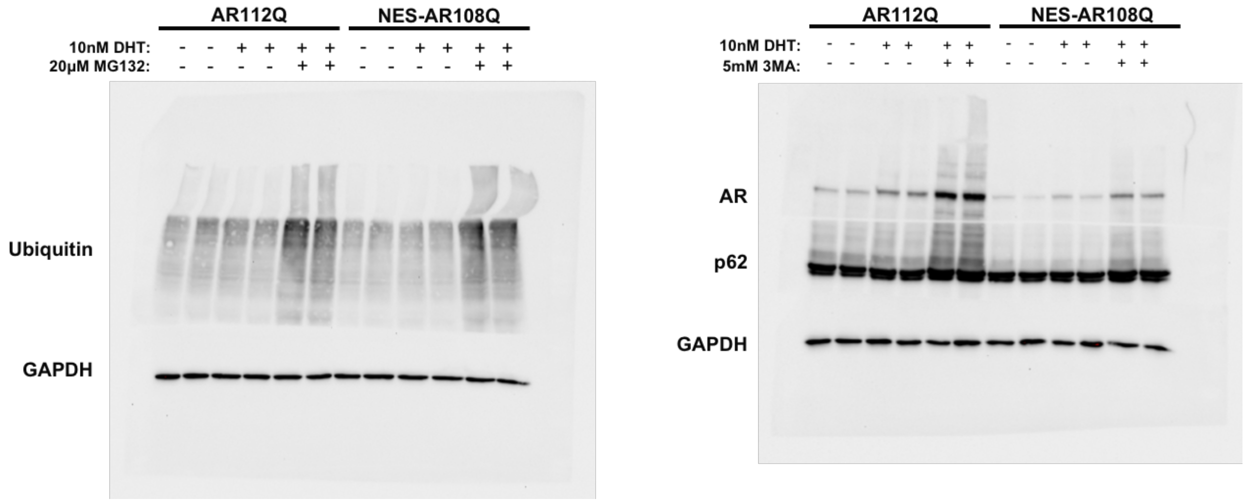

Figure S13. Full-length western blots corresponding to Figure S4A, B.

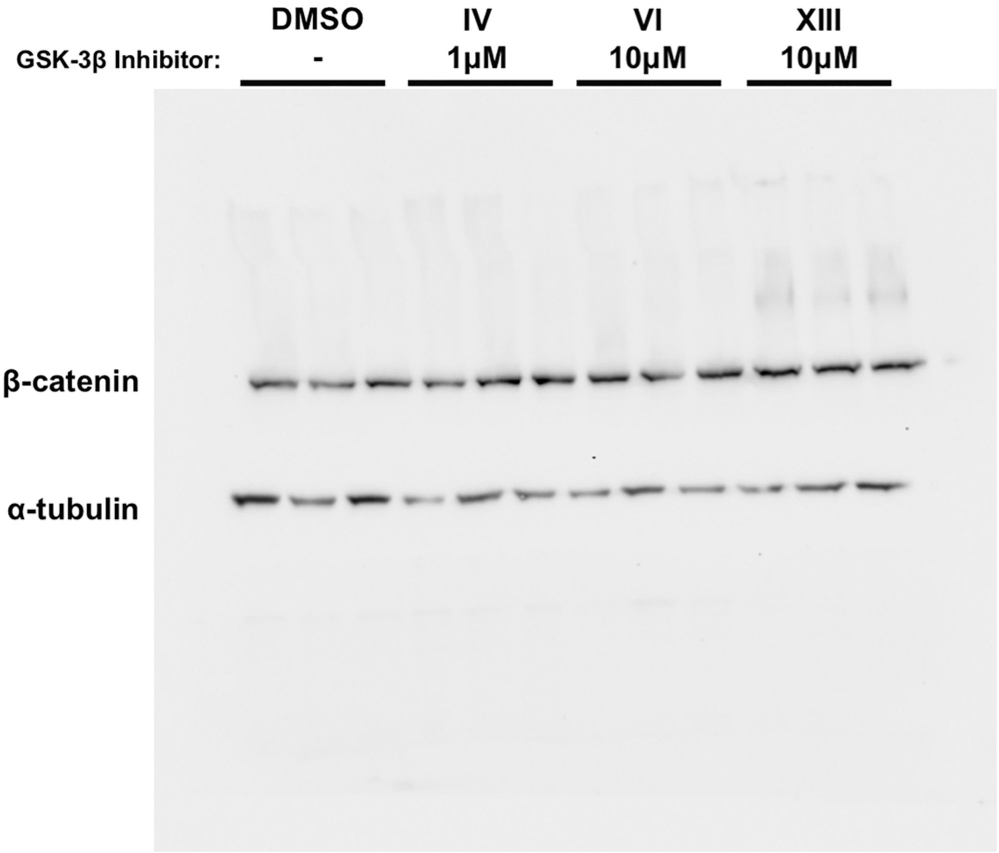

Figure S14. Full-length western blots corresponding to Figure S7.

Supplemental References:

1 Montie, H. L. *et al.* Cytoplasmic retention of polyglutamine-expanded androgen receptor ameliorates disease via autophagy in a mouse model of spinal and bulbar muscular atrophy. *Hum Mol Genet* **18**, 1937-1950, doi:10.1093/hmg/ddp115 (2009).
2 Datta, S., Snow, C. J. & Paschal, B. M. A pathway linking oxidative stress and the Ran GTPase system in progeria. *Mol Biol Cell* **25**, 1202-1215, doi:10.1091/mbc.E13-07-0430 (2014).
